# Quantitative analysis of handwriting kinematics in primary and lower secondary school children through a sensorized ink pen: A cross-sectional population-based study

**DOI:** 10.1371/journal.pdig.0001503

**Published:** 2026-07-23

**Authors:** Simone Toffoli, Linda Greta Dui, Stefania Fontolan, Francesca Lunardini, Milad Malavolti, Chiara Piazzalunga, Alice Donati, Cesare Cornoldi, Cristiano Termine, Simona Ferrante

**Affiliations:** 1 Department of Electronics, Information and Bioengineering, Politecnico di Milano, Milano, Italy; 2 Department of Medicine and Technological Innovation, Università degli Studi dell’Insubria, Varese, Italy; 3 Center for Clinical Neuroscience, Hospital los Madroños, Madrid, Spain; 4 Department of General Psychology, Univeristà di Padova, Padova, Italy; 5 ASST Sette Laghi - Struttura Complessa Neuropsichiatria Infantile e dell’Adolescenza, Varese, Italy; 6 LEARNLab, IRCCS Istituto Neurologico Carlo Besta, Milano, Italy; McGill University, CANADA

## Abstract

The assessment of handwriting is fundamental for identifying difficulties, which may have long-term negative consequences. However, standard evaluation typically focuses only on the final handwritten product. For this reason, Italian guidelines recommended supporting traditional evaluation with digital tools to also analyze the handwriting process. A sensorized ink pen used on paper was employed by over 700 students, ranging from first grade in Italian primary school to third grade in lower secondary school, to execute two tasks of the BVSCO-3, the gold standard for handwriting assessment. From sensorized ink pen data, handwriting indicators in the domains of Time, Force, Smoothness, Tilt, and Frequency were extracted. These indicators were then analyzed to examine their correlation with clinical scores, to model cross-sectional trends across grades, and to identify handwriting difficulties. The correlation analysis revealed significant relationships between the indicators and the clinical score, particularly for the Time domain. A cross-sectional statistical analysis showed that the indicators follow developmental trends compatible with handwriting learning curves reported in the literature: for many indicators, a performance plateau was reached in grade 3, from both motor and processing perspectives. Lastly, binary classification models successfully distinguished subjects with handwriting difficulties (based on BVSCO-3 results) from proficient writers. The sensorized ink pen allowed uncovering relevant characteristics of children’s handwriting process, while guaranteeing ecological data acquisition conditions. Its use could pave the way for a prompt identification of handwriting difficulties in school settings, thus facilitating an efficient referral to clinical services.

## Author summary

Learning to write is an important part of a child’s development, and persistent handwriting difficulties can affect school performance and self-confidence. Traditionally, handwriting is assessed by looking only at the final written text, which may miss important information about how the writing is produced. In this study, we explored whether a sensorized pen that writes on normal paper can provide useful additional insights into children’s handwriting. We worked with over seven hundred Italian primary and lower secondary school students from all grades, who completed standard handwriting tasks while using the sensorized pen. This tool allowed us to measure aspects of the writing process, such as how long writing takes, how much force is applied, how smooth the movements are, and how the pen is held. We found clear links between these measurements and clinical handwriting scores. Importantly, the digital indicators reflected well-known patterns of handwriting development, showing that many skills stabilize around grade 3. Our results also show that data from the digital pen can distinguish children with handwriting difficulties from proficient writers. We believe this approach offers an easy-to-use and natural way to support the identification of handwriting problems in schools, potentially improving access to clinical assessment and intervention.

## 1. Introduction

Even though typewriting has become widespread, teaching handwriting to children remains fundamental because of its positive effects on spelling, reading, and overall literacy [1–3]. To produce a meaningful written trace, high-level executive functions are activated, including the intention to write, the retrieval of orthographic and semantic knowledge from long-term memory, and the activation of appropriate motor schemes [4], [5], [6], [7]. The produced trace must be monitored through sensory-motor feedback, to correct possible mistakes [8]. Finally, several joints and muscles must be coordinated to manipulate the writing instrument. If the gesture is efficient, the cognitive load is reduced, allowing more resources to be allocated to composition [1,2,9–11].

But how do children learn to write? After a spontaneous phase of drawing figures [9], skills are acquired sequentially. Children first master general aspects of writing, including understanding its symbolic nature, and later develop language-dependent features [12]. This process is supported by formal education in primary school, which devotes considerable time to paper-and-pencil activities [13]. The literature agrees on the importance of the first three years of primary school for handwriting development [4,14–17], as handwriting quality improves the most during this period. Indeed, by 3^rd^ grade, children are expected to achieve automaticity in the gesture [4,15,18]. However, writing speed continues to increase throughout the school years and stabilizes at around 15 years of age [4,9,14,19].

Unfortunately, not all children follow this path. Some face handwriting difficulties, which are referred to as dysgraphia when they persist in absence of neurological or intelligence weaknesses [20]. Dysgraphia can occur with other neurodevelopmental disorders and may present differently across individuals. It typically results in slow and/or illegible handwriting, which makes it difficult for pupils to meet school demands [9] and negatively affects their wellbeing [21]. Reported consequences include frustration and lack of motivation, reduced self-esteem, academic and professional failure, emotional and relational problems [22]. These findings highlight the need for early identification of handwriting difficulties [5,10,23], along with the right intervention and support, to promote both academic success and personal wellbeing.

Several handwriting assessment methods have been proposed. Although they differ in the type and length of tasks (e.g., copying words, sentences, or paragraphs; dictation; or text production), they share a common focus on handwriting speed and quality [20,24,25]. Speed is measured as the number of letters produced within a given time interval or vice versa, while quality is assessed through visual inspection of size, slant, spacing, and overall appearance [25,26].

Despite being widely employed, these methods are time-consuming and suffer from poor inter-rater reliability [25,27,28]. Moreover, when the evaluation focuses only on the written product, it is challenging to evaluate the child’s motor performance, potentially overlooking important aspects of the writing process. The heterogeneity of the existing tests, coupled with their limitations, could explain the wide range of reported prevalence of handwriting difficulties, which varies from 5% to over 30% [5,29–31].

Digital technologies have enabled the analysis of the handwriting process, that is, the ensemble of kinematic and dynamic aspects that underlie the generation of the written trace [25,26]. Digitizers record the trajectory of the writing tool and the applied pressure, providing an objective description of movement across multiple domains. In the temporal domain, in-air (i.e., non-writing) movements have been shown to characterize both typical development [32] and impairments [33], as they reflect the time needed to elaborate the motor program. Smooth pen movements are a clear sign of automation, independent from handwriting quality and speed [34]. The number of inversions in the velocity profile (NIV) has been proposed as a measure of smoothness: higher values of NIV indicate less fluent gestures and have been observed in non-proficient writers [35,36]. Jerk has also been used as a descriptor of movement fluency and motor control [26,37]. Another parameter is the neuromotor noise, which reflects the inability to suppress involuntary high frequency oscillations of the upper limb and has been correlated with poor handwriting performance [38]. The characterization of kinematic power spectra using thresholding-based methods has been adopted to distinguish children with and without dysgraphia by Asselborn et al. [36,39]. Children with dysgraphia tend to show broader spectral distributions, indicative of a suboptimal motor control. Non-proficient handwriting has also been associated with variable force values, which may contribute to reduced movements fluency [40,41]. The analysis conducted in [42] confirmed that 3^rd^ grade of primary school represents a turning point, followed by a second, slower phase of handwriting development. Another important finding is the decrease in the variability of these quantitative measures with age and schooling [43], reflecting the progressive stabilization of motor schemes.

The critical role of technology for handwriting assessment has also been emphasized in Italy. In 2021, national clinical guidelines [44] fostered the integration of digital devices into the traditional handwriting assessment. These guidelines also highlighted the relevance of studying handwriting learning curves, which support evidence-based identification of difficulties.

The present work proposes a novel approach for the quantitative characterization of the graphic gesture tested in Italian students. By using a sensorized ink pen (SIP) designed to write on ordinary paper, the aim was to capture key features of the graphic gesture in an ecological setting, without altering children’s usual writing activity. Handwriting data were collected while more than 700 students performed a standardized handwriting speed task [45], and a set of indicators describing temporal, motor, and dynamic aspects of writing were extracted.

The aims of this study were threefold. First, to assess the validity of indicators, i.e., whether they are meaningfully associated with established clinical handwriting scores. Second, to evaluate their ability to model cross-sectional trends across grades. Third, to evaluate their ability to identify children with and without handwriting difficulties. The results showed that all three aims were achieved, demonstrating that process-based handwriting measures obtained with a sensorized ink pen can support both the characterization of handwriting development and the identification of difficulties in the school context.

## 2. Results

### 2.1. Participants and dataset

A total of 707 students were recruited during the 2022/2023 and 2023/2024 school years from four public primary schools and one public lower secondary school in the province of Varese (Lombardy, Italy), and one private lower secondary school in the province of Novara (Piedmont, Italy). Table 1 summarizes their characteristics for each grade, from 1^st^ to 5^th^ grade of primary school and from 1^st^ to 3^rd^ grade of lower secondary school, and time point of data collection (T1 = December for 2^nd^ grade and February for 1^st^ grade; T2 = May). For clarity, 1^st^, 2^nd^, and 3^rd^ grades of lower secondary school are hereafter referred to as 6^th^, 7^th^ and 8^th^ grade, respectively.

**Table 1:** Sample Characteristics. Age and Z Scores are given as mean ± standard deviation. N.A.: not available; N.C.: not considered. Laterality Coherence: “YES” (Y) when the pupil’s dominant hand, eye and foot were ipsilateral, “NO” (N) otherwise. As for nationality, mother tongue, and language at home – the main language spoken at home: Italian (ITA) or any other (other), since children attended classrooms held in italian. For preferred allograph, “B” and “C”: block letters and cursive.

Variable	1–T1	1–T2	2–T1	2–T2	3	4	5	6	7	8
Age (years)	6.64 ± 0.34	6.86 ± 0.33	7.39 ± 0.37	7.86 ± 0.37	8.62 ± 0.32	9.66 ± 0.32	10.70 ± 0.41	11.67 ± 0.33	12.68 ± 0.33	13.63 ± 0.25
Sex (F; M)	46; 66	45; 64	57; 48	57; 51	58; 40	54; 44	69; 46	48; 32	21; 34	22; 20
Handedness (R; L; N.A.)	102; 9; 1	101; 8; 0	94; 11; 0	95; 11; 1	86; 9; 3	84; 11; 3	105; 10; 0	73; 7; 0	52; 3; 0	34; 7; 1
Laterality coherence (Y; N; N.A.)	39; 73; 0	37; 72; 0	35; 70; 0	36; 71; 1	46; 52; 0	49; 49; 0	43; 72; 0	53; 26; 1	32; 23; 0	24; 18; 0
Nationality (ITA; Other; N.A.)	92; 20; 0	89; 20; 0	75; 30; 0	76; 31; 1	81; 17; 0	78; 20; 0	83; 32; 0	N.A.	N.A.	N.A.
Mother tongue (ITA; Other; N.A.)	98; 13; 1	96; 13; 0	102; 3; 0	103; 4; 1	94; 4; 0	90; 5; 3	102; 10; 3	71; 9; 0	53; 2; 0	41; 1; 0
Language at home (ITA; Other; N.A.)	97; 14; 1	95; 14; 0	85; 20; 0	86; 21; 1	94; 4; 0	90; 5; 3	102; 10; 2	70; 10; 0	52; 3; 0	41; 1; 0
Preferred allograph (B; c; N.A.)	102; 9; 1	100; 9; 0	64; 40; 1	68; 40; 0	40; 58; 0	51; 47; 0	67; 47; 0	47; 33; 0	34; 21; 0	31; 11; 0
UNO_B – Z	−0.46 ± 0.56	−0.12 ± 0.82	0.17 ± 0.77	−0.07 ± 0.73	0.08 ± 1.14	−0.19 ± 0.89	−0.26 ± 1.24	0.16 ± 0.96	−0.26 ± 0.89	−0.17 ± 0.91
UNO_B – NR; R	105; 6	N.C.	103; 2	N.C.	87; 11	90; 7	95; 19	75; 5	49; 5	40; 2
NUM_B – Z	−0.44 ± 0.75	−0.31 ± 0.88	−0.17 ± 0.64	−0.35 ± 0.72	−0.18 ± 0.92	−0.63 ± 0.82	−0.65 ± 1.47	−0.19 ± 0.97	−0.70 ± 0.90	−0.55 ± 0.88
NUM_B – NR; R	106; 5	N.C.	102; 1	N.C.	92; 6	83; 15	80; 34	69; 10	46; 9	36; 6
UNO_c – Z	N.A.	−1.88 ± 0.51	N.A.	−1.58 ± 0.75	−1.20 ± 0.75	−1.50 ± 0.79	−1.38 ± 0.88	−1.46 ± 1.07	−1.39 ± 0.83	−1.63 ± 1.02
UNO_c – NR; R	N.A.	17; 88	N.A.	51; 55	59; 35	53; 40	62; 45	41; 38	28; 25	15; 27
NUM_c – Z	N.A.	-2.02 ± 0.48	N.A.	-1.55 ± 0.83	-1.30 ± 0.74	-1.65 ± 0.89	-1.45 ± 1.26	-1.41 ± 1.24	-1.51 ± 1.06	-1.83 ± 1.12
NUM_c – NR; R	N.A.	14; 91	N.A.	53; 50	57; 37	37; 55	51; 55	41; 36	26; 28	16; 25
Missing Data	2	8	2	7	8	12	21	1	2	2

This work considers two tasks of the BVSCO-3 battery that evaluate handwriting speed [45]. In both tasks, children were asked to write on a sheet of paper using the SIP for one minute. The tasks consisted of writing either sequence of the word “UNO” (“ONE” in Italian) or the number sequence as words (“UNO DUE TRE…”, i.e., “ONE TWO THREE…” in Italian). Children were instructed to write as quickly as possible and adopted both block letters and cursive allographs. A total of four tasks were available: UNO and NUM in block letters (UNO_B and NUM_B), and in cursive (UNO_*c* and NUM_*c*). Table 1 reports the Z scores and the number of subjects classified as not at risk (Z score > -1.5, NR) and at risk (Z score ≤ -1.5, R) of having handwriting difficulties. First-grade students showed the lowest Z scores in both UNO_*c* and NUM_*c*. The smaller number of cursive samples compared to block letters is due to some students being unable to perform the tasks in cursive. The number of missing data, due to acquisition errors or the impossibility of testing some subjects, is reported in the “Missing Data” row. As shown in Table 1, block letters was the preferred allograph, particularly in 1^st^ (98.23% at T1 and 91.74% at T2) and 8^th^ grades (73.81%). This preference was associated with significantly worse Z scores in cursive among children who used both allographs (see S1 Table). This outcome was consistent across grades and tasks, even in 3^rd^ grade, where most students preferred cursive. It may be explained by the later introduction of cursive in the school curriculum, which varies across schools in timing and teaching methods.

### 2.2. SIP indicators validity

**Fig 1:**
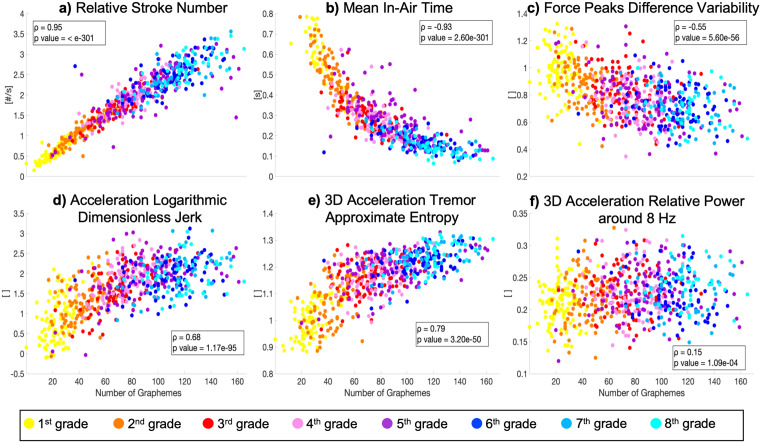
Correlations between number of graphemes and indicators in the NUM_B task. Scatterplots for NUM_B, with correlation coefficient (ρ) and p value. Yellow: 1^st^ grade T1. Orange: 2^nd^ grade T1. Red: 3^rd^ grade. Pink: 4^th^ grade. Purple: 5^th^ grade. Blue: 6^th^ grade. Light blue: 7^th^ grade. Cyan: 8^th^ grade.

**Fig 2:**
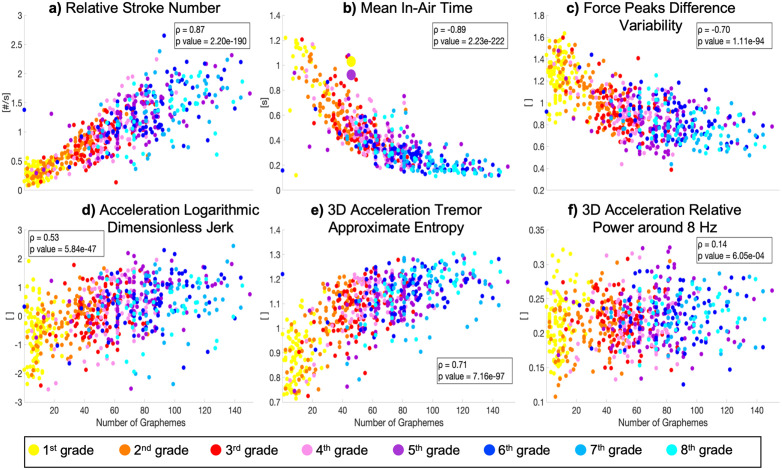
Correlations between number of graphemes and indicators in the NUM_*c* task. Scatterplots for NUM_*c*, with correlation coefficient (ρ) and p value. The color code is the same as in Fig 1.

To assess the validity with respect to the BVSCO-3, SIP indicators were grouped across grades (choosing T1 for 1^st^ and 2^nd^ graders in block letters tasks) and their correlation with the number of correctly written graphemes was computed. For clarity, only a subset of indicators is presented in Figs 1 and 2 for NUM_B and NUM_*c*, respectively (results for UNO_B and UNO_*c* are reported in S1 Fig and S2 Fig). The selected indicators represent different aspects of handwriting performance. In the temporal domain, the *Relative Stroke Number* (panel a) is the indicator most closely related to the BVSCO-3 score. The *Mean In-Air Time* (b) reflects the time spent planning the movements and is therefore interpreted as a measure of cognitive load. In the Force domain, *Force Peaks Difference Variability* (c) captures how consistently force is applied during writing. Movement smoothness is quantified by the *Acceleration Logarithmic Dimensionless Jerk* (d), with lower values indicating less fluent and more irregular movements. The *3D Acceleration Tremor Approximate Entropy* (e) describes the regularity of small, high-frequency oscillations of the hand, which are related to motor control stability. Finally, the *3D Acceleration Relative Power around 8 Hz* (f) represents the contribution of specific frequency components in the movement, providing information on neuromotor control. Correlation results are reported as Spearman’s Rank coefficients (ρ), as the data were not normally distributed, with the associated p-values. The complete results are provided in S2 Table. The analysis revealed significant and moderate-to-strong correlations in the domains of Time, Force, Smoothness, and Kinematic Oscillations, in line with the expectations, and their correlations were generally weak. Overall, the Time domain exhibited the highest percentage of strong correlations, followed by the Smoothness domain (see S3 Fig).

### 2.3. SIP indicators cross-sectional trends

The indicators were analyzed using statistical tests for independent samples. When significant differences were found, post-hoc comparisons were performed using Dunn–Šidák correction to understand how indicators differed across primary and lower secondary school. For block letters, the first time point was selected for 1^st^ and 2^nd^ grades.

**Fig 3:**
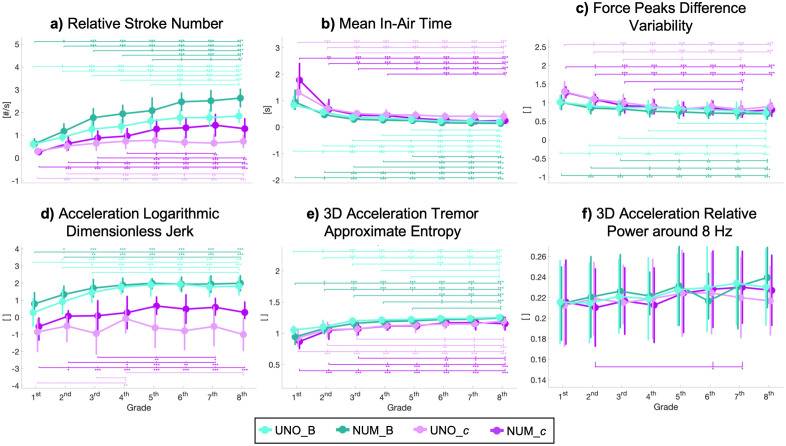
SIP indicators cross-sectional trend. For each indicator and grade, the dot represents the indicator central value (either the mean or the median), while the vertical bars represent its variability (either ± standard deviation or 25^th^ - 75^th^ percentiles). Cyan: UNO_B. Teal: NUM_B. Pink: UNO_*c*. Purple: NUM_*c*. The horizontal lines display the pairwise statistically significant differences between grades. The p value is encoded by the asterisk: ‘*’, p value < 0.005; ‘**’, p value < 0.001; ‘***’, p value < 0.0001.

Fig 3 displays the results for the same indicators presented in the correlation analysis. The complete results are provided in the S1 Data. Five out of the six indicators showed significant differences across grades.

**Fig 4:**
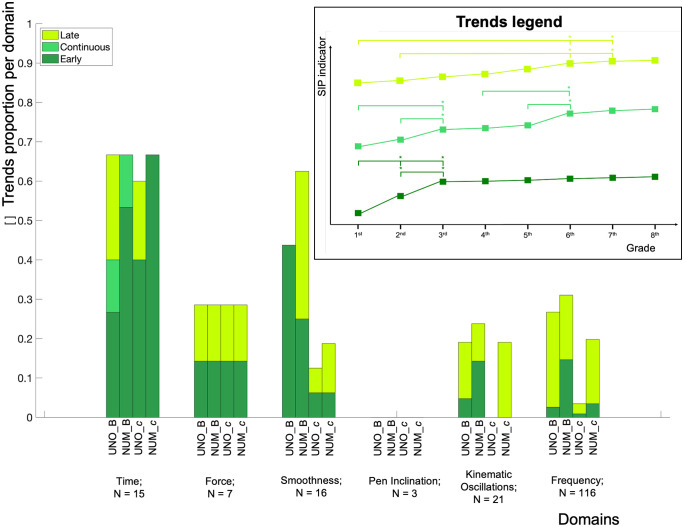
SIP cross-sectional trend percentage by domains and tasks. Dark green: Early development; Green: Continuous development; Light green: Late development. Only indicators with some statistical significance in the post hoc are colored in the bars, normalized according to the total number of indicators in the domain (N). In the Trends legend panel, squares indicate the mean/median value of a hypothetical indicator computed at each grade; horizontal lines with asterisks represent significant differences between grades. The representation is only for explanation purpose and does not correspond to real distribution or significance in statistical testing.

Fig 4 provides an overview of all indicators. For each domain and each BVSCO-3 task, the percentage of indicators following a given cross-sectional trend is reported. Three types of trends were identified: 1) Early development, where performance reaches a plateau by 3^rd^ grade; 2) Continuous development, where significant differences between adjacent grades are found both in the early primary school and between the final years of primary school and lower secondary school; 3) Late development, where significant improvements compared to 1^st^ and 2^nd^ grades emerge only from 5^th^ grade onward. Examples of these three patterns are presented in the *Trends legend* panel of Fig 4.

Overall, the Time domain revealed the highest percentages of indicators following a cross-sectional trend, with a prevalence of early development. It was also the only domain in which continuous development was identified. The Force domain showed a balanced distribution across tasks, while in the Smoothness domain the cross-sectional trends were more evident in block-letter tasks. The remaining domains included fewer indicators that followed a cross-sectional trend.

### 2.4. Identification of handwriting difficulties

Binary classification models were built to distinguish pupils at risk (R) of handwriting difficulties (defined as Z score ≤ -1.5) from those not at risk (NR), with separate models built for each task. Data from all grades were grouped together to develop a single age-independent model for each task.

For cursive tasks, samples from 1^st^ grade were excluded, as many pupils had not yet mastered this writing form. This is reflected in their lowest Z scores among all grades and the high proportion of R children (see Table 1).

The analysis pipeline is described in the Methods section, Fig 8. Table 2 shows the classification performance on the test set, presented as mean (standard deviation) across multiple test sets: five-fold nested cross-validation for AdaBoost (AB) and random forest (RF), five train-test splits for the tabular foundation model (TabPFN). Information on the training strategy is also provided. For comparison, the results of a dummy classifier, always classifying the majority class, are reported. Model performance was evaluated on the R group using recall, precision, f1 score and area under the precision-recall curve.

**Table 2:** Classification Metrics and Training Strategy of the Best Performing Models. Model: Classification algorithm (AB = Adaboost; RF = Random forest, TABPFN = Tabular foundation model). PRC AUC: Area under the precision-recall curve. PROT INFO: 1 If the demographic information was included in the input features. UP-SAMPLER: Up-sampling strategy (SVMSMT = SVM Smote). FS: 1 If feature selection was applied in the training.

model	accuracy	recall	specificity	precision	f1 score	prc auc	prot info	up- sampler	FS	
UNO_B: NR = 644; R = 57										
dummy	0.92	0	1	0	0	0.08				
RF	0.94 (0.01)	0.57 (0.17)	0.98 (0.02)	0.72 (0.18)	0.61 (0.09)	0.43 (0.10)	0	svmsmt	1	
**AB**	**0.94 (0.03)**	**0.64 (0.10)**	**0.96 (0.03)**	**0.67 (0.23)**	**0.64 (0.15)**	**0.46 (0.19)**	**1**	**smote**	**0**	
TabPFN	0.93 (0.03)	0.66 (0.16)	0.95 (0.04)	0.57 (0.17)	0.59 (0.14)	0.40 (0.17)	0	no	0	
NUM_B: NR = 613; R = 86										
dummy	0.88	0	1	0	0	0.12				
RF	0.93 (0.02)	0.83 (0.13)	0.95 (0.02)	0.70 (0.09)	0.75 (0.09)	0.60 (0.13)	0	svmsmt	1	
**AB**	**0.94 (0.01)**	**0.79 (0.06)**	**0.96 (0.02)**	**0.77 (0.10)**	**0.77 (0.05)**	**0.63 (0.07)**	**1**	**no**	**1**	
TabPFN	0.92 (0.02)	0.59 (0.20)	0.97 (0.02)	0.74 (0.10)	0.64 (0.14)	0.49 (0.13)	1	svmsmt	1	
UNO_c: NR = 309; R = 266										
dummy	0.54	0	1	0	0	0.46				
RF	0.79 (0.02)	0.76 (0.06)	0.82 (0.03)	0.78 (0.03)	0.77 (0.03)	0.70 (0.03)	0	no	1	
AB	0.81 (0.04)	0.78 (0.05)	0.84 (0.04)	0.81 (0.04)	0.79 (0.04)	0.73 (0.05)	0	no	1	
**TabPFN**	**0.82 (0.04)**	**0.77 (0.03)**	**0.87 (0.06)**	**0.84 (0.06)**	**0.80 (0.04)**	**0.75 (0.05)**	**0**	**no**	**0**	
NUM_c: NR = 281; R = 288										
dummy	0.51	1	0	0.51	0.67	0.51				
RF	0.81 (0.03)	0.82 (0.06)	0.81 (0.08)	0.82 (0.06)	0.82 (0.03)	0.76 (0.04)	0	no	0	
AB	0.81 (0.01)	0.82 (0.06)	0.80 (0.06)	0.81 (0.05)	0.81 (0.13)	0.75 (0.02)	1	no	0	
**TabPFN**	**0.84 (0.04)**	**0.81 (0.04)**	**0.86 (0.05)**	**0.86 (0.04)**	**0.83 (0.04)**	**0.79 (0.04)**	**0**	**no**	**0**	

**Fig 5:**
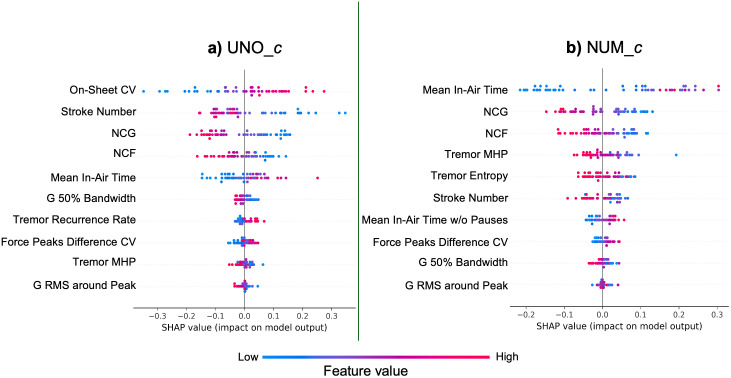
Shapley values of the ten most important features for each model classification. a), UNO_c; b), NUM_c. Each dot represents a subject, colored according to the magnitude of features value (purple = high, blue = low). Deviation from the vertical 0 line represents the extent to which each feature contributes to the classification towards R (right) or NR (left). Some indicator names have been modified for visualization purposes: On-Sheet CV = On-Sheet Time Variability; NCG = Angular Velocity Relative Number of Changes; NCF = Force Relative Number of Changes; G 50% Bandwidth = Angular Velocity Median Frequency; Force Peaks Difference CV = Force Peaks Difference Variability; Tremor MHP = 3D Acceleration Tremor Mean Harmonic Power; G RMS around Peak = Median Angular Velocity RMS around the Peak; Tremor Entropy = 3D Acceleration Tremor Approximate Entropy.

**Fig 6:**
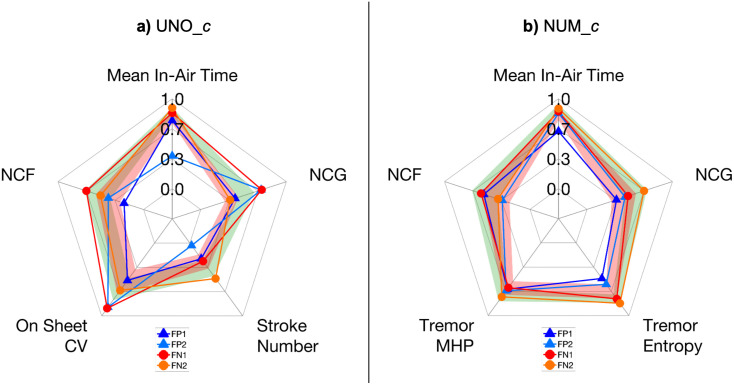
SIP indicators polar plots with misclassified subjects. a) UNO_*c*; b) NUM_*c*. The green area represents the interquartile range of the indicators in the NR group, while the red area does the same for the R group. The correspondence with the formal indicators names is: NCG = *Angular Velocity Relative Number of Changes*; NCF = *Force Relative Number of Changes*; On Sheet CV = *On Sheet Time Variability*; Tremor Entropy = *3D Acceleration Tremor Approximate Entropy*; Tremor MHP = *3D Acceleration Tremor Mean Harmonic Power*. Blue triangles and red/orange circles locate representative subjects on each indicator axes.

**Fig 7:**
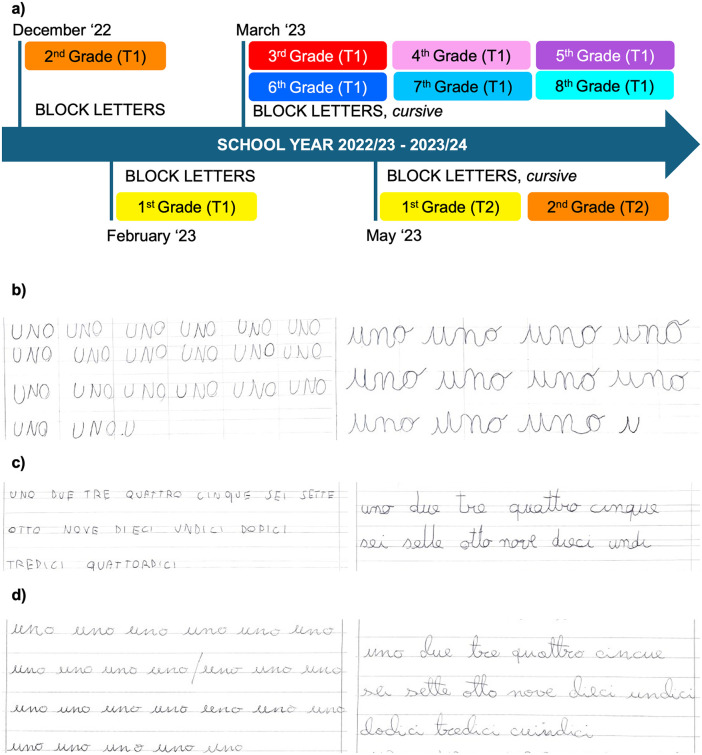
Protocol and writing samples. a) timeline of BVSCO-3 UNO and NUM acquisitions, with details on the adopted allographs for each grade; b) UNO_B and UNO_c samples from a first grader; c) NUM_B and NUM_c samples from a third grader; d) UNO_c and NUM_c samples from a fourth grader.

**Fig 8:**
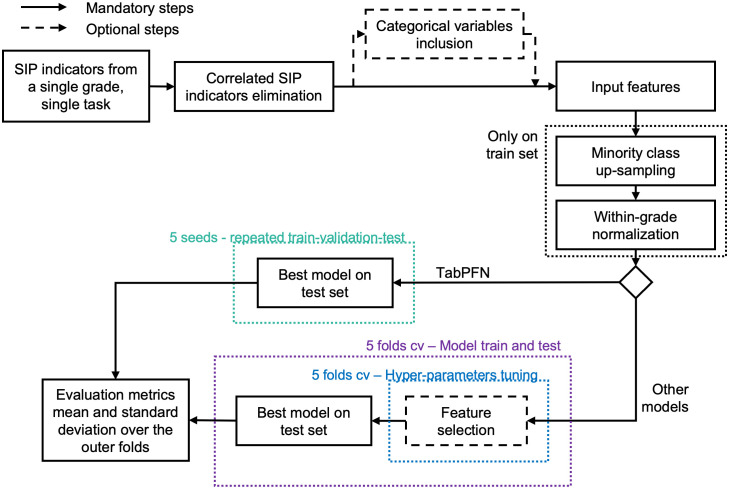
Pipeline adopted for the development of the binary classification models. The pipeline was performed for each task and algorithm. Solid arrows and blocks refer to steps that are always performed in building the model. Dashed lines and boxes refer to steps that could or could not take place. The purple dotted box contains the blocks included in the nested cross-validation outer loop, while the blue dotted box contains the blocks included in the inner loop. It is worth noting that the “Minority class up-sampling” block includes the option of avoiding up-sampling.

The SHAP explainability technique [46] was applied on the best-performing models, after retraining them on the full dataset, to identify which SIP indicators contributed most to the model classifications. This analysis was conducted only on models trained on cursive samples, as models based on block letters showed lower performance. It was verified that reducing the number of features and subjects for the explainability analysis did not affect model performance, as the results remained within the 95% confidence interval of the mean performance obtained from the full analysis pipeline. Fig 5 shows the ten most relevant indicators for both UNO_*c* and NUM_*c*.

The results were consistent across cursive tasks. Eight of the ten most relevant indicators were shared between UNO_*c* and NUM_*c*, namely: *Stroke Number*, *Angular Velocity Relative Number of Changes* (NCG), *Force Relative Number of Changes* (NCF), *Mean In-Air Time*, *Angular Velocity Median Frequency* (G 50% Bandwidth), *Force Peaks Difference Variability* (Force Peaks Difference CV), *3D Acceleration Tremor Mean Harmonic Power* (Tremor MHP), *Median Angular Velocity RMS around the Peak* (G RMS around Peak). The direction of the effects was also consistent across tasks: when low values of a given indicator were associated with the R group in UNO_*c*, the same pattern was observed in NUM_*c*.

SHAP explainability was further exploited to analyze misclassified subjects. The polar plots in Fig 6 display two false positives (blue triangles) and two false negatives (red circles) for the models based on cursive samples. These misclassified subjects shown for the UNO_*c* model are not necessarily the same as those shown in the NUM_*c* model. The five most relevant indicators are displayed in normalized units (ranging from 0 to 1). For visualization purposes, all indicators were transformed so that higher values (closer to 1) correspond to the NR class. In at least two out of the five indicators, misclassified subjects showed values that were not consistent with their true group, which may explain the incorrect predictions.

## 3. Discussion

The acquisition of efficient handwriting is fundamental for children. Therefore, the prompt identification of weaknesses is crucial. The shortcoming of the Italian procedures lie in its timing of assessment [44], which typically occurs in the 3^rd^ grade of primary school and is often preceded by long waiting lists. For this reason, Italian guidelines recommend the introduction of digital solutions to enable a more efficient detection of handwriting difficulties, allowing timely intervention. This work represents a first step in this direction. Handwriting speed tasks were administered to more than 700 students from Italian primary and lower secondary schools using a sensorized ink pen. The proposed approach is compatible with both the classroom environment and the traditional pen-and-paper format used in standard assessments, ensuring ecological validity.

### 3.1. SIP indicators validity

Given the use of a novel instrument, its validity was first evaluated. The *Relative Stroke Number* (i.e., the rate of stroke production) – as done for scoring – correlation coefficients were close to 1 in block-letter tasks (Fig 1A, S2 Table). This is because, in block letters, each grapheme typically corresponds to a single stroke detected by the SIP. Deviations from a perfect correlation can be attributed to occasional errors in letter formation. In contrast, greater variability was observed in cursive writing, reflecting the writer’s personal style. In UNO_*c*, the correlation coefficient decreased to 0.68 (see S2 Fig, panel a), likely reflecting differences in writing strategies. Skilled writers tend to produce the entire word in a single stroke, whereas beginners and less proficient writers may lift the pen between letters. This can be inferred from S2 Fig, panel a, where three distinct trends emerge from 4^th^ grade onward, corresponding to different rates of pen lifts. A similar but less pronounced effect was observed in NUM_*c* (ρ = 0.87, Fig 2A), as some words inherently require pen lifts. Strong negative correlations were found for *Mean In-Air Time* (Figs 1B and 2B) across all tasks, in agreement with previous studies [27,33,47]. This indicates that proficient writers achieve higher speed mainly by reducing the time spent between strokes, rather than by writing faster within each stroke. Regarding Force, lower variability in the difference between consecutive force peaks (*Force Peaks Difference Variability*, Figs 1C and 2C) was associated with better performances. This suggests that proficient writers apply pressure more consistently, which is necessary for achieving motor automaticity [41,48]. This effect was more evident in cursive tasks, where longer strokes make force patterns more clearly observable. Movement smoothness, quantified by the *Acceleration Logarithmic Dimensionless Jerk* (Figs 1D and 2D), was higher in students who wrote more quickly, indicating smoother movements [49]. However, this relationship was weaker in UNO_*c* (ρ = 0.15). Again, this may reflect differences in writing strategies, as the smoothness of a word written in a single movement differs from that of a word composed of separate letter strokes. Pen tilt was not significantly associated with handwriting speed in the recruited sample, in line with previous research [36,50]. The *3D Acceleration Tremor Approximate Entropy* (Figs 1E and 2E) showed a clear pattern: as writing speed increased, high-frequency oscillations in the acceleration signal become increasingly stochastic. This suggests that proficient writers in primary and lower secondary school already show signs of handwriting maturation, as unpredictability in human kinematics is associated with mature motor control [9]. However, the relative power in frequency bands centered around 5 Hz (S2 Table) and 8 Hz (Figs 1F and 2F), and the bandwidths (*Acceleration Tremor First Principal Component 90% Bandwidth Frequency*), have been reported to increase in children with handwriting difficulties [36,38,39]. In this study, these indicators showed weak but positive correlations with the BVSCO-3 score, contrary to the expectations. In the literature, spectral analysis is typically performed on velocity signals recorded from digitizer screens [38]. In the present study, the lack of spatial trajectory information prevented the adoption of this approach, which relies on the identification of a reference movement profile for the estimation of neuromotor noise. Moreover, the sampling frequency of the SIP is lower than that commonly used in digitizers (50 Hz vs 100/200 Hz, respectively). This limited the achievable frequency resolution, particularly when analyzing short signal segments such as individual strokes. Indeed, handwriting strokes are often very brief (typically below 0.5 s, and even shorter in block letters), making stroke-level spectral analysis unreliable under these conditions. To overcome these constraints, spectral features were computed on the acceleration signal over the entire task, including in-air movements. While this approach ensures adequate frequency resolution, it introduces discontinuities related to pen lifts and recontacts, which are not directly associated with neuromotor noise and may influence the spectral estimates. These methodological differences may explain some discrepancies with the literature [36,38,39].

Overall, the validity analysis highlights the multifaceted nature of the kinematic aspects of handwriting fluency. Writing speed depends on several interacting components, including automatized motor planning, smooth movements, and fine control of the pen-paper interaction. Therefore, although the BVSCO-3 tasks are evaluated clinically based on speed alone, they capture multiple underlying aspects of the handwriting process. This was particularly evident in UNO_*c*, where relying solely on the number of strokes measured by the SIP can be misleading.

### 3.2. SIP indicators cross-sectional trends

In handwriting speed, measured by *Relative Stroke Number* (Fig 3A), significant improvements were found across all grades. In UNO_B, a pattern of continuous cross-sectional development was found, whereas in the other tasks the development was early. This does not imply that later grades did not show better performance than the earlier ones; rather, these differences were less consistent. The trend can be explained by the *Mean In-Air Time* (Fig 3B). Its marked decrease between grades (continuous in block letters and early in cursive) likely reflects the progressive optimization of motor planning processes [5,8]. This aspect is still developing in the first two grades of primary school: longer in-air time indicates the increased effort required to plan words. This was evident in the NUM tasks of 1^st^ graders, where converting the familiar phonemic (spoken) representation of numbers into the graphemic (written) one increased the cognitive demand [29,51]. A similar pattern was observed in a preliminary study on a smaller sample [52]. Overall, the Time domain showed the highest percentage of SIP indicators following grade-related patterns consistent with learning curves, mainly characterized by early development. Interestingly, it was the only domain in which continuous development was observed, in line with the literature stating that handwriting speed increases up to 15 years of age [19].

Improvements in speed are also linked to differences in motor execution. In the Force domain, variability in force peaks decreases with age (Fig 3C), suggesting improved control of the interaction with the writing surface, after an initial phase of exploration (late development in block letters, early in cursive) [23,41]. Instead, the force amplitude did not reveal a consistent trend (see Supplementary Data). Previous studies report mixed findings: some found no or negligible age-related effects [49] while others reported an increase [43]. Gesture Smoothness (*Acceleration Logarithmic Dimensionless Jerk*) was another key factor, confirming previous findings [49]. Although smoother execution was observed in older children, as reported in [42], the results differed depending on the allograph. In block letters, smoothness followed an early cross-sectional trend, supporting its role as an indicator of handwriting maturity. In cursive, however, no clear cross-sectional trend was observed, possibly due to the influence of individual writing styles (see for example the drop in NUM_*c* between grade 7 and 8 in Fig 3D). Notably, the Smoothness domain clearly differentiated the two allographs (Fig 3D). No significant grade effect was observed for the Tilt domain, consistent with the validity analysis. The regularity of high frequency oscillations (*3D Acceleration Tremor Approximate Entropy*, Fig 3E) followed a cross-sectional trend, towards more irregular (stochastic) patterns, occurring early in block letters and later in cursive. Regarding spectral analysis of these oscillations [38], significant results were observed only in NUM_c (Fig 3F). In general, the Frequency domain showed fewer indicators with significant cross-sectional trend, with a prevalence of late development. When significant effects were found, higher relative power in frequency bands associated with neuromotor noise and broader bandwidths (i.e., frequency intervals containing 90% of the spectral power) were found with increasing age. These findings differ from part of the previous literature [36,39,50], which suggests that improved motor control should reduce neuromotor noise. However, given the differences in signal acquisition and processing described above, direct comparison should be made with caution. In particular, the inclusion of in-air movements and the absence of trajectory-based normalization may lead to spectral features that reflect a combination of motor control and task execution strategies, rather than neuromotor noise alone. Alternatively, the observed increase in higher-frequency components may reflect a maturation-related expansion of motor control dynamics, approaching patterns seen in healthy adults [53], rather than a simple increase in neuromotor noise as traditionally interpreted.

Overall, SIP indicators in the domains of Time, Force, Smoothness, and Kinematic Oscillations described cross-sectional trends consistent with known handwriting learning curves. Significant changes were observed not only from the 1^st^ to the 3^rd^ grade of primary school, but also between the final years of primary school and lower secondary school. These changes reflect both improved cognitive processing and refinement of fine motor skills, supported by the substantial time (30–60%) devoted to writing activities in school [4,15]. Consistent with previous studies, 3^rd^ grade emerged as the time point when handwriting becomes more automatic. This plateau should not be interpreted as a halt in development, but rather as the stabilization of a consistent handwriting gesture. Indeed, these indicators continue to show a cross-sectional improvement beyond this stage, often showing further improvements compatible with late developmental trends.

### 3.3. Identification of handwriting difficulties

In studying handwriting difficulties, students were divided into two groups: at risk (R) and not risk (NR), based solely on their Z score. The classification results were highly influenced by the allograph. In block-letter tasks, the dataset was highly imbalanced, with the R group representing 8.1% and 12.3% of the total dataset in UNO_B and NUM_B, respectively. This resulted in average f1 scores of 0.64 and 0.77, even after applying data augmentation during training. Although these results were better than the dummy classifier, they were lower than those obtained with cursive tasks. For this reason, explainability analysis was not performed for block letters.

In cursive tasks, after excluding 1^st^ grade, the two target classes were more balanced, leading to improved and more stable classification performance. For both tasks, the best-performing model was TabPFN. It achieved an average f1 score of 0.80 in UNO_*c* and 0.84 in NUM_*c*. SHAP analysis showed a high level of consistency between the two models. Among the ten most relevant indicators, three belonged to the Time domain, two to Force, one to Smoothness, three to Kinematic Oscillations and one to Frequency, confirming the multifaceted nature of handwriting difficulties [36,54]. Eight indicators were shared between the two models and showed the same relationship with the target classes. In the Time domain, the R group was associated with a lower number of produced strokes (*Stroke Number*). Although it may seem intuitive, in cursive there is no one-to-one correspondence between strokes and letters, as shown in the validity analysis. Nevertheless, this simple measure remains informative for distinguishing poor from proficient handwriting. This suggests that handwriting difficulties in this sample are mainly related to reduced speed. In turn, low handwriting speed appears to result from a non-optimized programming of the motor schema. This is supported by higher values of *Mean In-Air Time* (and *Mean In-Air Time without Pauses* in NUM_*c*) found in R subjects, rather than slowness in the actual execution of the strokes. In UNO_*c*, temporal variability during on-paper execution (*On-Sheet Time Variability*) was the most important feature, in line with the validity analysis of UNO_*c*. This suggests that at-risk writers struggle to maintain a consistent strategy when repeatedly writing the same word, leading to irregular timing patterns. In the Force domain, a distinct pattern emerged: subjects in the R group showed fewer changes in the direction of force application (low *Force Relative Number of Changes*) than the NR group, but greater variability in force amplitude (high *Force Peak Difference Variability*), in accordance with the validity analysis. Rather than smoothly modulating pressure, these writers appear to adjust force in a reactive manner during movement. For Smoothness and Kinematic Oscillations, the models attribute handwriting difficulties to reduced modulation and lower amplitude in angular velocity (*Angular Velocity Relative Number of Changes* and *Median Angular Velocity RMS around the Peak*). These features are important to proficiently write in cursive, which requires continuous and well-coordinated curved movements. The regularity of high frequency oscillations also played a relevant role. In the R group, tremor patterns were more regular and predictable, as indicated by higher *Tremor Recurrence Rate* in UNO_*c* and lower *3D Acceleration Tremor Approximate Entropy* in NUM_*c.* This result is in line with the validity analysis, where higher entropy (i.e., more variability) was associated with better performance and increasing age. Therefore, high entropy values can be interpreted as a sign of handwriting proficiency. In the Frequency domain, the R group showed lower values, specifically in the bandwidth containing 50% of the spectral power of the angular velocity signal. While this result differs from some previous findings, it is consistent with the methodological considerations discussed above and should be interpreted in light of the specific signal acquisition and processing approach adopted in this study.

The SHAP analysis highlighted that handwriting difficulties are characterized by multiple interacting features of the writing process, which could be used to design personalized training programs. It also revealed heterogeneous patterns in misclassified subjects (Fig 5). For example, in UNO_*c* (Fig 5A), FP2 was misclassified because of atypical features in the temporal domain (*On-Sheet Time Variability*, *Mean In-Air Time, Stroke Number*), while FN1 showed values compatible with the NR group in several indicators (*Mean In-Air Time*, *On-Sheet Time Variability*, relative number of changes of both force and angular velocity). In NUM_c (Fig 5B), FP1 was similar to the R group in several indicators (*Mean In-Air Time*, *3D Acceleration Tremor Approximate Entropy* and *Angular Velocity Relative Number of Changes*), while FN2 showed the opposite pattern.

Overall, the classification results for cursive tasks were promising. However, direct comparison with the literature is not straightforward, since most studies focus on group-level differences between children diagnosed with dysgraphia and controls [36,39,55]. Another important aspect is the distribution of the Z scores used to define the target classes. In both UNO_*c* and NUM*_c*, average Z scores were lower than -1 across all grades, i.e., the recruited children performed below normative expectations of the BVSCO-3. This was expected for the 1^st^ and 2^nd^ grade at T2, since the normative data are referred to the children’s favorite allograph, and most of the students in this work preferred block letters (91.74% and 62.96%). Moreover, cursive is introduced later in the curriculum, which may explain poorer performance. However, this pattern was also observed in higher grades, where students were expected to be more familiar with cursive and chose their favorite allograph in a balanced way. The way in which cursive is introduced in the educational program may have influenced these outcomes. Just to give a snapshot, only 27 and 37 samples had a Z score greater than zero (i.e., the average value expected by the normative data of the BVSCO-3) in UNO_*c* and NUM_*c*, respectively. As a result, the classification models had limited exposure to high-performing samples, reducing their ability to learn patterns associated with average or above-average performance. Importantly, the collected data can support future studies comparing typically developing students with peers diagnosed with dysgraphia, enabling direct comparisons with the existing literature.

### 3.4. Limitations

The participants were recruited from schools in the same geographical area (Northern Italy), which may limit the generalizability of the findings. Differences in educational practices, handwriting instruction, and language across regions or countries may influence both handwriting performance and the associated process-based indicators. To produce more robust and generalizable results, future studies should include multi-site data collection across diverse educational and cultural contexts. Despite this limitation, the relatively large sample size and the inclusion of multiple grade levels provide a solid basis for exploratory analysis.

Expanding recruitment could also allow to gather more balanced data, better reflecting the distribution of the BVSCO-3 normative data. Although up-sampling strategies were employed to mitigate class imbalance, this issue still influenced model performance, as reflected by the lower F1 scores observed in UNO_B task. Class imbalance may also reduce the reliability and generalizability of the models, especially when applied to populations with different prevalence rates of handwriting difficulties. Therefore, the reported classification performance should be interpreted with caution, and future studies should consider larger and more balanced datasets, as well as alternative strategies specifically designed for imbalanced learning. Another limitation was the exclusion of first graders in the cursive classification models, due to their scarce proficiency with the allograph.

Besides these problems, performances could have also been altered by the larger size of the SIP with respect to standard pens. The data acquisition tool is one of the main differences from previous studies. Unlike digitizers, the SIP does not record the written trace. For this reason, trace-related indicators could not be computed, and incorrectly written characters cannot be excluded from the analysis. This limitation affects the possibility to link process-based indicators to correct letters. It also impacts the analysis of neuromotor noise, which in the literature is typically computed from the written trace only. Although the SIP allows the identification of on-paper and in-air moments through the force sensor, it cannot determine which strokes correspond to individual letters. Consequently, this study focuses on the motor components of graphomotor fluency only, without capturing spatial organization, letter formation accuracy, or visual-motor integration. Future development should aim to reconstruct the written trace from signals [56,57], enabling a more comprehensive analysis.

A longitudinal approach would also allow a better understanding of developmental trajectories and would be necessary to assess both the predictive validity of the proposed indicators for the early detection of handwriting difficulties and the within-subject evolution of handwriting skills over time. Indeed, the grade-related patterns observed in this study are derived from cross-sectional comparisons. While they are consistent with learning curves reported in the literature, they do not provide direct evidence of individual development. For this reason, the sensorized ink pen cannot yet be considered a screening tool. To improve this point, the data of third to eighth graders presented in the study should also be compared with those of subjects clinically diagnosed with dysgraphia.

Concerning cross-sectional trends, the analyses did not explicitly account for the hierarchical structure of the data (e.g., students nested within classes or schools). This may have influenced the observed variability. Future studies should adopt hierarchical or mixed-effects modeling approaches to better account for data dependency and provide more robust estimates of grade-related effects.

Lastly, after the development of a proper method to present the results of the analysis, the utility and usability of the entire system should be investigated in both teachers and clinicians.

## 4. Conclusions

Recent Italian guidelines claimed the urgency of complementing traditional dysgraphia assessment with objective analysis of the handwriting process to increase its accuracy and reliability, emphasizing the role of technological tools. This is particularly relevant for Italian students, as standardized digital norms are not yet available. In this study, we addressed this gap by providing a comprehensive quantitative evaluation of handwriting performance in a large sample of Italian primary and lower secondary school students. The results obtained with the use of the SIP were promising, while maintaining ecological data acquisition conditions. The SIP indicators demonstrated their validity with respect to the clinical standard for handwriting speed assessment and revealed developmental patterns in line with the literature. Lastly, the classification algorithms based on cursive tasks achieved satisfactory performances on real-world data collected in the relevant scenario. These findings provide the basis of digital norms for the assessment of handwriting kinematics in Italian students. The SIP can be used directly in school without altering usual writing activities, and the newly collected data can be analyzed to detect deviations from expected cross-sectional trends, highlighting the domains of difficulty for each child. Therefore, the SIP approach shows potential as a tool for a large-scale, digital, preclinical screening of handwriting difficulties in school environments, possibly supported by remote-monitoring solutions [58]. Its adoption would enable targeted referral to clinical services, ensuring that only children who truly need intervention are assessed, and enabling more personalized treatment strategies. Moreover, data collected during real-world use of the system can be exploited to further refine the classification models, in an adaptive AI framework, thus increasing their robustness and generalizability over time.

## 5. Methods

### 5.1. Ethics statement

The study complied with all relevant ethical regulations and was approved by the Ethical Committee of Politecnico di Milano (n. 30/2022). Written informed consent was obtained from the parent/guardian of each participant under 18 years of age before admission to the study.

### 5.2. Participants

Pupils from the five grades of Italian primary school and the three grades of lower secondary school were recruited. Students were excluded if diagnosed with any disorder, either of motor, cognitive or neurological nature, that could affect their handwriting performance. A minimum of 85 participants per grade was targeted for the five primary school grades, as handwriting skills evolve most rapidly during these years [14,42]. The sample size was determined based on recommendations for an adequate and conservative sample size when collecting normative data with potentially non-normally distributed variables [59].

### 5.3. Protocol

Participants underwent the assessment individually in a quiet room in their school; the child seated at a desk, under the supervision of an operator, who recorded the participant’s characteristics.

Concerning demographics and physical characteristics, the information collected was relevant as the literature reports association between some of them (sex, laterality) with handwriting problems [5]:

- Sex assigned at birth, either male or female (no other options were allowed).
- Dominant hand, either right or left, as observed when participants started to write (ambidextrous tendency was not considered due to its rarity).
- Dominant eye, either right or left, assessed asking the participants to look into a telescope-shaped sheet and recording the eye used for the task [60].
- Dominant foot, either right or left, assessed by asking the participants to kick a ball [60].

Additional participants’ characteristics may have impacted the Italian familiarity, which is important for the execution of the handwriting tasks included in the protocol:

- Preferred allograph, either block letters or cursive.
- Mother tongue, either Italian or other, as reported by the participant. This meant the first language learnt at birth.
- Language spoken at home, either Italian or other, as reported by the participant. This meant the language mainly used to communicate with parents and siblings.
- Nationality, either Italian or other, was available from the school office.

The protocol included handwriting tasks performed on a sheet of paper designed to match the templates commonly used in classroom activities. For 1^st^ and 2^nd^ graders, the template consisted of double rows (height: 0.7 cm top, 0.5 cm bottom), to support learning the differences between upper and lowercase letters. For 3^rd^ graders, the bottom row was reduced to 0.3 cm to facilitate cursive practice. From 4^th^ grade onward, a single-row template was used (1 cm).

The tasks were drawn from the “handwriting speed” section of the BVSCO-3 (“Batteria per la Valutazione clinica della Scrittura e della Competenza Ortografica”) battery [45], the gold standard in Italy for assessing writing skills in primary and lower secondary school children. All tasks must be performed as quickly as possible, within one minute. Instructions, start and stop commands were provided by the operator. Before starting, children were shown a printed example of the task. To ensure they understood the instructions, they were allowed to practice a few graphemes. The samples were removed from their sight before data collection began. Pupils first wrote the word “UNO” (“ONE” in Italian) repeatedly, and then wrote the sequence of numbers as words (e.g., “UNO DUE TRE…”, i.e., “ONE TWO THREE…”), up to the highest number they could reach. The two tasks (UNO and NUM in the following) were first performed adopting the child’s favorite allograph (cursive or block letters). After a pause to prevent fatigue, UNO and NUM were repeated with the other allograph.

The timing of the sessions followed the BVSCO-3 protocol. First and second graders were assessed twice: 1^st^ graders in February (1 – T1) and May (1 – T2), 2^nd^ graders in December (2 – T1) and May (2 – T2). They executed UNO and NUM in cursive only at T2, due to their limited proficiency in mastering the allograph at T1. Pupils from 3^rd^ grade on performed the protocol once in March. Fig 7 displays the acquisition timeline, detailing the tasks performed at each time point (“_B” stands for block letters, “_*c*” for cursive), and some handwritten samples Fig 7.

### 5.4. Sensorized ink pen

The sensorized ink pen (SIP) developed by Lunardini et al. [61] was used to perform the handwriting tasks. Within the 3D-printed case, the pen ink cartridge refill and the electronic components are housed. The SIP is equipped with a 9 degrees-of-freedom inertial measurement unit (IMU), featuring 3D linear accelerometer, gyroscope, and magnetometer enabling the recording of the handwriting kinematics. A force piezoelectric sensor registers the normal force exerted on the pen tip. An embedded flash memory stores the data recorded by the sensors, a total of 11 signals: three-axis acceleration, angular velocity and magnetic field, the force and the timestamp. The work by Lunardini et al. [61] reported strong validity against reference measurement and solid test-retest reliability when writing with the SIP at different ages. The signals are digitized at a sampling frequency of 50 Hz. The data package can be transferred offline to other devices through the low energy Bluetooth (BLE) module included in the electronics. In this study, a commercial Android tablet served for the purpose via a custom app, that manages authentication (avoiding improper usage), data acquisition and upload to a private server physically hosted at Politecnico di Milano.

### 5.5. Data analysis

#### 5.5.1. BVSCO-3 tasks scoring.

Adhering to the manual, the BVSCO-3 tasks were scored manually by trained operators. A raw score was assigned, corresponding to the total number of correctly written graphemes. A grapheme was counted as correct if it was correctly formed, regardless of its orthographic accuracy. For example, if a pupil writes “UMO” instead of “UNO” and the letter “M” is written correctly, it was included in the score. Errors in the numerical sequence (e.g., “UNO, DUE, QUATTRO”, i.e., 1-2-4, instead of “UNO, DUE, TRE”, i.e., 1-2-3) were not considered in the scoring. The raw score was converted into a Z score, using BVSCO-3 normative data, specific for each task, time point and grade. Importantly, these normative data refer to the favorite writing style (allograph) adopted by the children during the development of the test. To compare the performance between block letters and cursive, paired-samples statistical tests were performed (paired t-test for normally distributed data, Wilcoxon signed rank test otherwise). The analysis was conducted in MATLAB R2023b, considering the allograph as independent variable and the number of graphemes as dependent variable.

#### 5.5.2. Computation of SIP indicators.

The signals recorded by the SIP were elaborated in MATLAB R2023b, following the procedure described in [62,63] to extract 178 indicators characterizing handwriting performance across different domains. The indicators extracted from the SIP modelled process-based kinematic aspects of handwriting fluency. However, graphomotor fluency is a multidimensional construct that also includes spatial organization, letter formation accuracy, and visual–motor integration, which are not directly measured by the SIP. A selection of representative indicators is described hereafter for each domain.

In the Temporal domain, 15 indicators were extracted. These included the *Relative Stroke Number* [#/s], i.e., the number of strokes produced per second (a proxy of writing speed), the *Mean In-Air Time* [s] and the *In-Air Time Variability* representing the average time and variability of pen lifts between consecutive strokes, respectively. The same was done for the time spent on sheet (*Mean On-Sheet Time* [s] and *On-Sheet Time Variability* []), which reflect the duration and stability of pen contact with the writing surface.

The Force exerted on the writing surface was described by 7 indicators, like the *Force Relative Number of Changes* [#/s], which quantifies how frequently the applied force increases or decreases (i.e., force modulation during writing), the *Force Peaks Difference* [arbitrary units] and the *Force Peaks Difference Variability* [], representing the magnitude and variability of force fluctuations between consecutive extrema.

A pool of 16 indicators characterized the Smoothness of the handwriting gesture. The logarithmic dimensionless jerk was extracted from acceleration and angular velocity (*Acceleration Logarithmic Dimensionless Jerk* [] and *Angular Velocity Logarithmic Dimensionless Jerk* []) This measure reflects movement smoothness, with higher values indicating more abrupt and less coordinated motor control.

The *Stroke Mean Tilt* [deg], *Stroke Tilt Variance* [deg^2^] and *Stroke Tilt Variability* [] quantify the mean, variance and coefficient of variation of the SIP angle with respect to the vertical direction (90 deg with the SIP held vertically). These indicators describe the stability and consistency of pen orientation during writing.

Kinematic Oscillations were measured with 21 indicators. For example, the same strategy adopted for the force signal allowed obtaining the *Angular Velocity Peaks Difference* [deg/s] and the *Angular Velocity Peaks Difference Variability* []. The predictability of the acceleration high frequency oscillations, which can be considered as tremor, was computed with the approximate entropy (*Acceleration Tremor Approximate Entropy* []). Lower entropy values indicate more predictable oscillations, whereas higher values reflect more irregular and mature motor patterns.

The Frequency domain was described by 116 indicators. Thirty-six of them described neuromotor noise, i.e., involuntary or less controlled motor activity potentially affecting handwriting quality [38]. Such indicators related to spectral power distribution in predefined frequency bands: both bands related to pathological tremors [64] (voluntary and dyskinetic motion, Parkinson’s disease, essential and physiological tremor) and neuromotor noise (3 Hz wide bands, centered around 2, 5, 8 and 11 Hz) were investigated. For example, the *3D Acceleration Relative Power in the Band of Physiological Tremor* falls in the former category, while the *3D Acceleration Relative Power around 5 Hz* belongs to the latter.

Then, 45 indicators characterized spectral power distribution according to a thresholding strategy. The width of the frequency interval in Hz, starting from 0 Hz, containing the 50% (e.g., *3D Acceleration Median Frequency*) of the spectral power [39] was computed. This measure provides an estimate of how movement energy is distributed across frequencies. The *3D Acceleration Sorted Median Frequency* was extracted with a similar approach, after sorting the power spectra in descending order.

Lastly, 35 indicators related to different characteristics of the power spectra peak. The frequency (e.g., *3D Acceleration Spectral Peak Frequency*) and amplitude (e.g., *3D Acceleration Spectral Peak Amplitude*) were retained, together with the Outlier Level (e.g., *3D Acceleration Outlier Level*) and the Amplitude Modulated Outlier Level (e.g., *3D Acceleration Amplitude Modulated Outlier Level*), which quantify how prominent the peak is compared to the overall spectrum. Power concentration around the spectral peak was included as well, by computing the width in Hz of the frequency interval, centered on the peak frequency, containing 50% (e.g., *3D Acceleration Peak Power Concentration 50%*) of the total power. All frequency indicators were computed from spectral distribution with a frequency resolution equal to 0.1 Hz.

The SIP indicators were then analyzed with three different aims. The first and second analysis were conducted in MATLAB R2023b using a significance level of 5%. The Lilliefors test was employed to assess whether data were normally distributed. For the block letters tasks, T1 data were used for 1^st^ and 2^nd^ grade.

### 5.6. Aim i) – SIP indicators validity

For each task, indicator validity was assessed through a univariate correlation analysis with the BVSCO-3 score. To account for age-related effects, data from all grades were combined. Therefore, the results reflect overall associations across the developmental span rather than grade-specific relationships.

Given this approach, the raw BVSCO-3 score was selected as independent variable, whilst SIP indicators were considered as dependent variable. For each indicator, the Pearson’s correlation coefficient (for normally distributed data) or the Spearman’s rank correlation coefficient (for non-normally distributed data) was computed according to data distribution. Correlation strength was interpreted as weak (|r| ≤ 0.3), moderate (0.3 < |r| < 0.7), or strong (|r| ≥ 0.7).

### 5.7. Aim ii) – SIP indicators cross-sectional trend

Cross-sectional trends in handwriting were assessed by statistically comparing SIP indicators across grades, considering indicators as dependent variables and grade as the independent variable, for each task. ANOVA was used in case of normal distributions, while the Kruskal–Wallis test was applied otherwise. When significant differences were detected, post-hoc comparisons were performed using Dunn–Šidák correction.

According to the results of statistical tests, three patterns were considered (see Fig 4, Panel Trends legend): 1) Early development, when a performance plateau is reached within 3^rd^ grade; 2) Continuous development, when statistically significant differences are observed across all grades; 3) Late development, when differences emerge between early primary school and lower secondary school, but not within adjacent grades. For each BVSCO-3 task, the number of indicators falling within each category was computed.

### 5.8. Aim iii) – Identification of handwriting difficulties

The ability of SIP indicators to distinguish between good and poor handwriting performance was evaluated using binary classification models, developed in Python 3.11.11 using standard libraries (scikit-learn, imbalanced-learn) to improve reproducibility.

To achieve the widest dataset possible, data from all grades were combined. Given the intrinsic differences between block letters and cursive, separate analyses were carried out for the two allographs. Since cursive is usually introduced at the end of the 1^st^ grade, this grade was excluded from the cursive dataset for both UNO and NUM tasks. Instead, the block letters dataset includes all the eight grades.

The target classes were derived from the BVSCO-3 results. Pupils characterized by a Z score > -1.5 were considered as not at risk (NR) of handwriting difficulties in the specific task, while Z scores ≤ -1.5 were associated with a risk of handwriting difficulties (R). The threshold has a clinical significance, as it identifies children who may require attention in a screening context, whilst a more stringent threshold of -2 is used to diagnose dysgraphia, which is out of the scope of this work.

Before building the models, feature redundancy was reduced by removing highly correlated indicators. Specifically, indicators with a Spearman |correlation coefficient| ≥ 0.9 and p-value < 0.05 with at least one other indicator were removed [65]. For each correlated pair, the one occurring in second position was systematically removed, after ordering the domains in ascending relevance according to the literature. This pre-filtering step was aimed at reducing multicollinearity and improving model stability. Models were then developed using either only non-correlated SIP indicators or these indicators combined with the information collected from the students, to correct for potential confounders. This information was encoded as a binary variable for sex (0 for males), dominant hand (0 for right), lateral dominance (1 if hand, eye and foot dominances were ipsilateral), favorite allograph (0 for block letters), nationality (0 for Italy, 1 otherwise), mother tongue and main language spoken at home (0 for Italian, 1 otherwise).

Models were then trained differently, depending on the model itself. Indeed, three classification algorithms were evaluated: Random Forest (RF), AdaBoost (AB), and a tabular foundation model (TabPFN) [66]. These models were selected to represent complementary machine learning paradigms: ensemble-based methods (RF and AB), which are widely used for tabular biomedical data, and a tabular foundation model (TabPFN), which leverages pre-training on synthetic datasets to enable strong performance without task-specific hyperparameter tuning. This comparison allows evaluating whether recent foundation models provide advantages over more traditional approaches in this application domain.

As TabPFN is a foundation model, the model was directly trained and tested with a train-test random split strategy (80% train, 20% test), repeated five times for robustness, without hyper-parameter tuning. Instead, the other models were trained with a k-fold, stratified, nested cross-validation approach, considering 5 folds for the outer loop, where train and test sets were created, and 5 folds for the inner loop, responsible for the hyper-parameter tuning for the randomized search strategy. Specifically, for RF, the explored hyperparameters were the number of estimators [10,50,100], the criterion [‘gini’, ‘entropy’, ‘log_loss’], the minimum number of splits [2,5,10] and leaves [1,2,5], and the maximum number of features [‘sqrt’, ‘log2’, None]; for AB, the explored hyperparameters were the number of estimators [10,50,100], the learning rate [0.01, 0.03, 0.1], and the algorithm [‘SAMME’, ‘SAMME.R’].

Within the inner loop, different approaches for the up-sampling of the minority target class were explored: no up-sampling, SMOTE [67], Borderline SMOTE [68], SVM SMOTE [69] or ADASYN [70]. The mentioned up-sampling methods were imported from the imbalanced-learn library. For all the up-sampling models, the sampling strategy was set to the “minority” class, and random state was set to 42 to allow reproducible results. All up-sampling techniques were applied only to the training subsets within the cross-validation procedure, ensuring that no synthetic samples were introduced in the test data. This design prevents information leakage and allows a more reliable estimation of model performance under class imbalance conditions. At this stage a univariate feature selection step (SelectKBest, scikit-learn) was optionally applied within the cross-validation pipeline, ranking features based on their statistical association with the target variable, using k = [10,20,30,40,50]. This step was performed exclusively on the training data within each fold to avoid data leakage.

For all models, the input features were normalized leveraging within-grade standardization by subtracting the mean and dividing by the standard deviation computed on the train set only. This approach reduced grade dependency while avoiding data leakage. Given the potential class imbalance, the f1 score was used as optimization metric for non-foundation models. For each fold in the outer loop, the best model found in the inner loop was evaluated on the test set and the model accuracy, sensitivity/recall, precision, f1 score, and area under the precision recall curve (prc auc) were computed. The mean and standard deviation of the classification metrics over the five outer folds were then retained, together with the test results for TabPFN. The full pipeline is reported in Fig 8.

Once the best-performing model was identified, the SHAP explanation technique [46] was used to investigate the relevance of the indicators in the model classification. The best model was retrained on the whole dataset. To reduce computational costs, SHAP was applied on a subset of 10 features – selected as the most important considering the F-statistic of an ANOVA testing between the label and the indicator – and on 50 subjects. To obtain 50 representative subjects, instead of random sampling, they were selected as the centroid of a k-means clustering performed on the subjects themselves. The resulting analysis was reported in beeswarm plots, which highlight the contribution of each feature to model predictions.

Radar plots based on the 5 most relevant indicators were then created to show the behavior of the R/NR groups and to evaluate the trends in four misclassified subjects (two false positives and two false negatives) for each task.

## Supporting information

S1 Table Paired statistical comparison between the Z scores of block letters (B) and cursive (*c*) tasks of the BVSCO-3, for each grade. For 1^st^ and 2^nd^ grade, results are available at T2, given that cursive tasks were performed in that time point only. ‘N’ is the number of subjects considered for the statistical test, corresponding to the pupils who performed the tasks with both allographs. ‘Difference (*c* - B)’ is the difference between the Z score in the *c* task and the Z score in the B task, given as mean ± standard deviation when the difference was normally distributed and as median [25^th^ percentile; 75^th^ percentile] when the difference was not normally distributed. The last column represents the p value of the statistical test (paired sample t-test in case of normal distribution, Wilcoxon signed rank test otherwise).
(DOCX)

S2 Table Complete results of the correlation analysis. For each indicator, the measurement unit and the domain it characterizes are reported. The “Correlation Index” column reports the Spearman’s rank correlation between the indicator and the number of correctly written graphemes for UNO_B, NUM_B, UNO_*c* and NUM_*c* (the order is always the same), as in their combination at least one of them was always not normally distributed. The “p value” column contains the p value associated with the correlation value. The last column displays the intensity of the correlations: “Weak” if ≤ 0.3 in absolute value; “Moderate” if 0.3 < absolute value < 0.7; “Strong” if ≥ 0.7 in absolute value. Intensity and p value are not reported for non-significant correlations (p value ≥ 0.05).
(DOCX)

S1 Fig Correlations between number of graphemes and indicators in the UNO_B task. Scatterplots for UNO_B, with correlation coefficient (ρ) and p value. Yellow: 1st grade T1. Orange: 2nd grade T1. Red: 3rd grade. Pink: 4th grade. Purple: 5^th^ grade. Blue: 6th grade. Light blue: 7th grade. Cyan: 8th grade.
(TIF)

S2 Fig Correlations between number of graphemes and indicators in the UNO_*c* task. Scatterplots for UNO_*c*, with correlation coefficient (ρ) and p value. Yellow: 1^st^ grade T1. Orange: 2^nd^ grade T1. Red: 3^rd^ grade. Pink: 4^th^ grade. Purple: 5^th^ grade. Blue: 6^th^ grade. Light blue: 7^th^ grade. Cyan: 8^th^ grade.
(TIF)

S3 Fig Percentage of SIP indicators showing significant correlations with the number of correctly written graphemes, grouped by domain and task. Dark Green: Strong Correlation; Light Green: Moderate Correlation; Orange: Weak Correlation. The vertical axis is normalized according to the total number of indicators in the domain (N).
(TIFF)

S1 Data Complete results of the SIP Indicators Cross-sectional Trend analysis. The excel file is organized in different sheets, one for each investigated BVSCO-3 task. Each sheet is organized in the same way, the following columns being present: • *Indicator*, the indicator name, together with the measurement unit; • *domain*, the domain it belongs to; • *norm*, 0 if the indicator is normally distributed, 1 if not; • With X representing the grade from 1 to 8. ◦ *N_x* is the number of available samples for grade X; ◦ *central_x* is the indicator mean (*norm* = 0) or median (*norm* = 1); ◦ *var_x* is the indicator standard deviation (*norm* = 0) or interquartile range (*norm* = 1); ◦ *iqr_x* presents the indicator 25^th^ and 75^th^ percentiles, available only for *norm* = 1; *• p_morethanTwoSamples*, the p value of the statistical test comparing the distributions of all grades (ANOVA if *norm* = 0; Kruskal-Wallis if *norm* = 1). *• p_multcompare_x_y*, is the p value of the post-hoc pairwise comparison, conducted with the Dunn-Sidak’s methods, between the distribution of grade X and Y, with X different from Y. All the possible combinations of X and Y are present. The value is available only if *p_morethanTwoSamples* is lower than 0.05.
(XLSX)
