# Supplementary material for: Quantitative analysis of handwriting kinematics in primary and lower secondary school children through a sensorized ink pen: A cross-sectional population-based study
Source: PLOS Digit Health. 2026 Jul 23;5(7):e0001503. doi: 10.1371/journal.pdig.0001503 (PMC13395322; doi:10.1371/journal.pdig.0001503)
Supplement: S1 Table — For 1st and 2nd grade, results are available at T2, given that cursive tasks were performed in that time point only. ‘N’ is the number of subjects considered for the statistical test, corresponding to the pupils who performed the tasks with both allographs. ‘Difference (c - B)’ is the difference between the Z score in the c task and the Z score in the B task, given as mean ± standard deviation when the difference was normally distributed and as median [25th percentile; 75th percentile] when the difference was not normally distributed. The last column represents the p value of the statistical test (paired sample t-test in case of normal distribution, Wilcoxon signed rank test otherwise). (DOCX) [file pdig.0001503.s001.docx]

**S1 Table. Paired statistical comparison between the Z scores of block letters (B) and cursive (c) tasks of the BVSCO-3, for each grade.**

For 1st and 2nd grade, results are available at T2, given that cursive tasks were performed in that time point only. ‘N’ is the number of subjects considered for the statistical test, corresponding to the pupils who performed the tasks with both allographs. ‘Difference (c - B)’ is the difference between the Z score in the c task and the Z score in the B task, given as mean ± standard deviation when the difference was normally distributed and as median [25th percentile; 75th percentile] when the difference was not normally distributed. The last column represents the p value of the statistical test (paired sample t-test in case of normal distribution, Wilcoxon signed rank test otherwise).

| **Task** | **Grade (Time Point)** | **N** | **Difference (*c* - B)** | **p value** |
| --- | --- | --- | --- | --- |
| UNO | 1 (T2) | 104 | -1.41±0.63 | 2.66e-42 |
|  | 2 (T2) | 103 | -1.78±1.03 | 1.83e-32 |
|  | 3 (T1) | 94 | -1.32±1.02 | 7.51e-22 |
|  | 4 (T1) | 93 | -1.29±0.99 | 1.29e-21 |
|  | 5 (T1) | 107 | -1.19±1.04 | 4.53e-21 |
|  | 6 (T1) | 79 | -1.60±1.09 | 3.00e-21 |
|  | 7 (T1) | 53 | -1.12±0.96 | 2.20e-11 |
|  | 8 (T1) | 41 | -1.46±0.99 | 1.09e-11 |
| NUM | 1 (T2) | 104 | -1.58±0.66 | 1.67e-44 |
|  | 2 (T2) | 99 | -1.42±0.93 | 1.52e-27 |
|  | 3 (T1) | 94 | -1.14±0.91 | 6.23e-21 |
|  | 4 (T1) | 92 | -0.95 [-1.50; -0.55] | 6.08e-15 |
|  | 5 (T1) | 106 | -0.95±1.06 | 3.60e-15 |
|  | 6 (T1) | 77 | -1.24±1.00 | 3.56e-17 |
|  | 7 (T1) | 54 | -0.79±0.88 | 2.04e-8 |
|  | 8 (T1) | 41 | -1.29±1.08 | 2.06e-9 |
