# Supplementary material for: Quantitative analysis of handwriting kinematics in primary and lower secondary school children through a sensorized ink pen: A cross-sectional population-based study
Source: PLOS Digit Health. 2026 Jul 23;5(7):e0001503. doi: 10.1371/journal.pdig.0001503 (PMC13395322; doi:10.1371/journal.pdig.0001503)
Supplement: S2 Table — For each indicator, the measurement unit and the domain it characterizes are reported. The “Correlation Index” column reports the Spearman’s rank correlation between the indicator and the number of correctly written graphemes for UNO_B, NUM_B, UNO_c and NUM_c (the order is always the same), as in their combination at least one of them was always not normally distributed. The “p value” column contains the p value associated with the correlation value. The last column displays the intensity of the correlations: “Weak” if ≤ 0.3 in absolute value; “Moderate” if 0.3 < absolute value < 0.7; “Strong” if ≥ 0.7 in absolute value. Intensity and p value are not reported for non-significant correlations (p value ≥ 0.05). (DOCX) [file pdig.0001503.s002.docx]

**S2 Table. Complete results of the correlation analysis.**

For each indicator, the measurement unit and the domain it characterizes are reported. The ‘‘Correlation Index’’ column reports the Spearman’s rank correlation between the indicator and the number of correctly written graphemes for UNO_B, NUM_B, UNO_c and NUM_c (the order is always the same), as in their combination at least one of them was always not normally distributed. The “p value” column contains the p value associated with the correlation value. The last column displays the intensity of the correlations: “Weak” if ≤ 0.3 in absolute value; “Moderate” if 0.3 < absolute value < 0.7; “Strong” if ≥ 0.7 in absolute value. Intensity and p value are not reported for non-significant correlations (p value ≥ 0.05).

| **Indicator** | **Domain** | **Correlation**  **Index** | **p value** | **Intensity** |
| --- | --- | --- | --- | --- |
| Stroke Number [#] | Time | UNO_B 0.94 NUM_B 0.95 UNO_*c* 0.60 NUM_*c* 0.87 | < e-292 < e-292 3.91e-63 3.58e-195 | Strong Strong Moderate Strong |
| Relative Stroke Number [#/s] | Time | 0.94 0.95 0.58 0.86 | < e-292 < e-292 1.91e-59 2.20e-190 | Strong Strong Moderate Strong |
| Mean On-Sheet Time [s] | Time | -0.80 -0.77 -0.40 -0.74 | 4.58e-158 2.31e-140 3.05e-26 2.68e-111 | Strong Strong Moderate Strong |
| On-Sheet Time Variability [] | Time | -0.29 -0.21 -0.54 -0.20 | 5.10e-15 4.04e-08 3.73e-49 3.71e-07 | Weak Weak Moderate Weak |
| On-Sheet Ratio [] | Time | 0.30 0.48 0.22 0.27 | 2.17e-16 4.09e-42 3.18e-08 5.71e-12 | Moderate Moderate Weak Weak |
| Mean In-Air Time [s] | Time | -0.92 -0.93 -0.74 -0.89 | 4.07e-292 2.60e-301 5.28e-112 2.23e-222 | Strong Strong Strong Strong |
| In-Air Time Variability [] | Time | 0.32 0.16 -0.17 0.10 | 8.84e-18 3.28e-05 1.54e-05 1.22e-02 | Moderate Weak Weak Weak |
| Mean In-Air Time without Pauses [s] | Time | -0.92 -0.90 -0.61 -0.82 | 3.28e-286 4.57e-260 6.28e-66 1.93e-151 | Strong Strong Moderate Strong |
| In-Air Time Variability without Pauses [] | Time | 0.66 0.67 0.12 0.69 | 3.23e-89 4.49e-93 1.67e-03 5.28e-90 | Moderate Moderate Weak Moderate |
| Mean Pause Duration [s] | Time | -0.58 -0.69 -0.64 -0.70 | 1.83e-65 4.26e-98 1.47e-73 6.05e-93 | Moderate Moderate Moderate Moderate |
| Pause Duration Variability [] | Time | -0.52 -0.66 -0.61 -0.70 | 8.22e-50 1.00e-87 1.27e-67 2.11e-93 | Moderate Moderate Moderate Moderate |
| Pause Number [#] | Time | -0.60 -0.72 -0.66 -0.72 | 5.39e-69 4.29e-115 1.28e-80 1.59e-102 | Moderate Strong Moderate Strong |
| Relative Pause Number [] | Time | -0.60 -0.73 -0.66 -0.73 | 2.39e-70 3.94e-118 1.57e-82 5.60e-105 | Moderate Strong Moderate Strong |
| Air-Sheet Ratio without Pauses [] | Time | -0.29 -0.26 0.01 0.17 | 3.47e-15 2.22e-12  1.91e-05 | Weak Weak  Weak |
| Air-Sheet Ratio [] | Time | -0.42 -0.57 -0.29 -0.38 | 1.15e-30 1.23e-62 3.09e-14 2.67e-23 | Moderate Moderate Weak Moderate |
| Stroke Mean Force [arbitrary] | Force | -0.04 -0.07 0.02 0.01 |  |  |
| Mean Force [arbitrary] | Force | -0.01 -0.02 0.08 0.06 | 3.39e-02 | Weak |
| Stroke Force Variability [] | Force | 0.49 0.13 -0.10 0.03 | 8.98e-44 4.76e-04 8.56e-03 | Moderate Weak Weak |
| Force Overshoot [arbitrary] | Force | 0.15 0.11 0.04 0.05 | 6.94e-05 4.24e-03 | Weak Weak |
| Force Relative Number of Changes [#/s] | Force | 0.66 0.77 0.76 0.78 | 4.89e-89 1.11e-139 1.86e-121 8.58e-128 | Moderate Strong Strong Strong |
| Force Peaks Difference [arbitrary] | Force | 0.27 -0.01 0.04 0.10 | 5.43e-13   1.23e-02 | Weak   Weak |
| Force Peaks Difference Variability [] | Force | -0.40 -0.55 -0.60 -0.70 | 9.06e-29 5.60e-56 2.97e-63 1.11e-94 | Moderate Moderate Moderate Strong |
| Angular Velocity Relative Number of Changes [#/s] | Smoothness | 0.85 0.83 0.80 0.76 | 1.59e-198 3.64e-179 7.32e-144 1.80e-120 | Strong Strong Strong Strong |
| Acceleration Relative Number of Changes [#/s] | Smoothness | 0.50 0.46 0.55 0.48 | 6.89e-46 7.20e-38 1.00e-52 1.77e-38 | Moderate Moderate Moderate Moderate |
| Acceleration Logarithmic Dimensionless Jerk [] | Smoothness | 0.74 0.68 0.16 0.53 | 9.91e-122 1.17e-95 8.40e-05 5.84e-47 | Strong Moderate Weak Moderate |
| Angular Velocity Logarithmic Dimensionless Jerk [] | Smoothness | 0.70 0.50 0.19 0.55 | 2.29e-102 1.37e-44 1.39e-06 2.91e-51 | Moderate Moderate Weak Moderate |
| Stroke Angular Velocity Spectral Arc Length 10% [] | Smoothness | 0.70 0.50 0.31 0.58 | 1.81e-102 1.16e-44 1.09e-15 6.27e-59 | Moderate Moderate Moderate Moderate |
| Stroke Angular Velocity Spectral Arc Length 20% [] | Smoothness | 0.59 0.40 0.27 0.54 | 2.86e-66 5.93e-28 3.06e-12 7.37e-50 | Moderate Moderate Weak Moderate |
| Stroke Angular Velocity Spectral Arc Length 30% [] | Smoothness | 0.45 0.29 0.21 0.47 | 3.55e-36 1.03e-14 5.93e-08 9.00e-36 | Moderate Weak Weak Moderate |
| Stroke Angular Velocity Spectral Arc Length 40% [] | Smoothness | 0.34 0.18 0.14 0.38 | 9.42e-21 2.25e-06 5.83e-04 1.78e-23 | Moderate Weak Weak Moderate |
| Stroke Angular Velocity Spectral Arc Length 45% [] | Smoothness | 0.32 0.15 0.12 0.36 | 4.91e-18 4.66e-05 2.40e-03 4.48e-21 | Moderate Weak Weak Moderate |
| Stroke Angular Velocity Spectral Arc Length 50% [] | Smoothness | 0.23 0.05 0.06 0.28 | 1.57e-09   9.35e-13 | Weak   Weak |
| Angular Velocity Spectral Arc Length 10% [] | Smoothness | -0.25 -0.39 -0.30 -0.45 | 2.45e-11 3.45e-26 5.19e-15 4.33e-33 | Weak Moderate Moderate Moderate |
| Angular Velocity Spectral Arc Length 20% [] | Smoothness | -0.26 -0.41 -0.31 -0.46 | 2.43e-12 1.31e-29 1.70e-15 7.10e-35 | Weak Moderate Moderate Moderate |
| Angular Velocity Spectral Arc Length 30% [] | Smoothness | -0.26 -0.40 -0.30 -0.47 | 4.42e-12 3.84e-28 3.62e-15 9.86e-36 | Weak Moderate Moderate Moderate |
| Angular Velocity Spectral Arc Length 40% [] | Smoothness | -0.28 -0.41 -0.33 -0.49 | 2.69e-14 3.90e-30 3.00e-17 9.78e-39 | Weak Moderate Moderate Moderate |
| Angular Velocity Spectral Arc Length 45% [] | Smoothness | -0.28 -0.42 -0.33 -0.49 | 4.69e-14 1.74e-31 1.91e-17 7.62e-39 | Weak Moderate Moderate Moderate |
| Angular Velocity Spectral Arc Length 50% [] | Smoothness | -0.30 -0.46 -0.35 -0.53 | 2.38e-16 4.87e-38 5.51e-20 3.10e-46 | Moderate Moderate Moderate Moderate |
| Stroke Mean Tilt [deg] | Pen Inclination | 0.18 0.17 -0.02 0.07 | 1.86e-06 4.10e-06 | Weak Weak |
| Stroke Tilt Variability [] | Pen Inclination | -0.45 -0.15 -0.08 -0.14 | 4.21e-37 5.43e-05 4.49e-02 4.39e-04 | Moderate Weak Weak Weak |
| Stroke Tilt Variance [deg^2] | Pen Inclination | -0.40 -0.10 -0.11 -0.12 | 1.15e-27 8.56e-03 5.07e-03 2.96e-03 | Moderate Weak Weak Weak |
| Angular Velocity Peaks Difference [deg/s] | Kinematic Oscillations | -0.22 -0.27 -0.12 0.03 | 4.07e-09 8.63e-13 2.71e-03 | Weak Weak Weak |
| Angular Velocity Peaks Difference Variability [] | Kinematic Oscillations | -0.17 -0.29 -0.53 -0.50 | 8.23e-06 2.35e-15 2.38e-48 2.57e-41 | Weak Weak Moderate Moderate |
| Median Angular Velocity RMS around the Peak [deg/s] | Kinematic Oscillations | 0.38 0.40 0.43 0.47 | 7.21e-25 4.50e-28 8.03e-31 1.32e-36 | Moderate Moderate Moderate Moderate |
| Median Angular Velocity Amplitude Rate [deg/s^2^] | Kinematic Oscillations | -0.17 0.05 -0.13 0.13 | 6.57e-06  8.85e-04 7.64e-04 | Weak  Weak Weak |
| Maximum Angular Velocity RMS around the Peak [deg/s] | Kinematic Oscillations | 0.36 0.37 0.36 0.43 | 4.45e-23 3.07e-24 8.00e-21 1.09e-29 | Moderate Moderate Moderate Moderate |
| Maximum Angular Velocity Amplitude Rate [deg/s^2] | Kinematic Oscillations | -0.13 0.09 -0.14 0.14 | 4.36e-04 1.25e-02 5.03e-04 4.05e-04 | Weak Weak Weak Weak |
| 3D Acceleration Tremor Approximate Entropy [] | Kinematic Oscillations | 0.75 0.79 0.55 0.71 | 3.59e-126 3.20e-150 8.66e-51 7.16e-97 | Strong Strong Moderate Strong |
| 3D Acceleration Tremor Recurrence Rate [] | Kinematic Oscillations | -0.36 -0.51 -0.51 -0.57 | 2.81e-23 1.25e-47 2.20e-43 2.37e-56 | Moderate Moderate Moderate Moderate |
| 3D Acceleration Tremor Determinism [] | Kinematic Oscillations | -0.47 -0.57 -0.49 -0.60 | 8.95e-40 9.03e-63 1.54e-40 3.63e-63 | Moderate Moderate Moderate Moderate |
| 3D Acceleration Tremor Stability Index - Time EMD [Hz] | Kinematic Oscillations | -0.17 -0.08 -0.18 -0.14 | 3.71e-06 3.95e-02 3.28e-06 2.78e-04 | Weak Weak Weak Weak |
| 3D Acceleration Tremor Stability Index Threshold - Time EMD [Hz] | Kinematic Oscillations | -0.29 -0.27 -0.26 -0.24 | 7.39e-15 7.13e-13 4.99e-11 1.33e-09 | Weak Weak Weak Weak |
| 3D Acceleration Tremor Stability Index - Frequency EMD [Hz] | Kinematic Oscillations | -0.18 -0.09 -0.14 -0.07 | 1.46e-06 1.57e-02 4.86e-04 | Weak Weak Weak |
| 3D Acceleration Tremor Signal-to-Noise Ratio - EMD [dB] | Kinematic Oscillations | -0.11 -0.22 -0.08 -0.21 | 3.93e-03 2.27e-09  1.13e-07 | Weak Weak  Weak |
| 3D Acceleration Tremor Mean Harmonic Power - EMD [Log((mm/s^2^)^2^/Hz)] | Kinematic Oscillations | 0.37 0.40 0.40 0.47 | 5.98e-24 9.75e-28 2.38e-25 1.14e-35 | Moderate Moderate Moderate Moderate |
| 3D Acceleration RMS [mm/s^2^] | Kinematic Oscillations | 0.09 0.13 0.09 0.15 | 1.69e-02 4.27e-04 2.01e-02 1.59e-04 | Weak Weak Weak Weak |
| Angular Velocity RMS [deg/s] | Kinematic Oscillations | 0.19 0.24 0.12 0.22 | 2.37e-07 7.46e-11 2.55e-03 1.07e-08 | Weak Weak Weak Weak |
| 3D Acceleration Tremor Stability Index - Time [Hz] | Kinematic Oscillations | -0.23 -0.30 -0.08 -0.15 | 1.20e-09 1.80e-16  1.36e-04 | Weak Moderate  Weak |
| 3D Acceleration Tremor Stability Index Threshold - Time [Hz] | Kinematic Oscillations | -0.25 -0.26 -0.22 -0.21 | 2.76e-11 4.27e-12 2.14e-08 1.32e-07 | Weak Weak Weak Weak |
| 3D Acceleration Tremor Stability Index - Frequency [Hz] | Kinematic Oscillations | -0.17 -0.05 -0.12 -0.10 | 1.11e-05  2.54e-03 1.63e-02 | Weak  Weak Weak |
| 3D Acceleration Tremor Signal-to-Noise Ratio [dB] | Kinematic Oscillations | -0.35 -0.35 -0.17 -0.29 | 3.78e-21 1.15e-21 2.51e-05 6.07e-14 | Moderate Moderate Weak Weak |
| 3D Acceleration Tremor Mean Harmonic Power [Log((mm/s^2^)^2^/Hz)] | Kinematic Oscillations | 0.23 0.22 0.26 0.28 | 6.58e-10 2.31e-09 4.64e-11 3.21e-13 | Weak Weak Weak Weak |
| Median Angular Velocity Relative Power around the Peak [] | Frequency | -0.04 -0.22 0.05 -0.00 | 2.11e-09 | Weak |
| Maximum Angular Velocity Relative Power around the Peak [] | Frequency | -0.03 -0.16 0.07 0.01 | 1.45e-05 | Weak |
| 3D Acceleration Relative Power in the Band of Voluntary Motion [] | Frequency | -0.44 -0.45 -0.36 -0.46 | 2.63e-35 4.37e-36 2.08e-20 1.65e-34 | Moderate Moderate Moderate Moderate |
| 3D Acceleration Relative Power in the Band of Dyskinetic Motion [] | Frequency | 0.07 0.03 0.20 0.16 | 6.23e-07 5.38e-05 | Weak Weak |
| 3D Acceleration Relative Power in the Band of Parkinsonian Tremor [] | Frequency | 0.54 0.56 0.35 0.50 | 2.14e-53 5.02e-58 2.99e-20 7.41e-41 | Moderate Moderate Moderate Moderate |
| 3D Acceleration Relative Power in the Band of Physiological Tremor [] | Frequency | 0.42 0.47 0.35 0.49 | 5.54e-32 5.73e-39 5.63e-20 2.21e-39 | Moderate Moderate Moderate Moderate |
| 3D Acceleration Relative Power around 2Hz [] | Frequency | -0.42 -0.54 -0.21 -0.41 | 5.98e-32 1.74e-53 1.44e-07 1.28e-26 | Moderate Moderate Weak Moderate |
| 3D Acceleration Relative Power around 5Hz [] | Frequency | 0.35 0.42 0.17 0.33 | 5.24e-22 1.43e-31 1.46e-05 2.89e-17 | Moderate Moderate Weak Moderate |
| 3D Acceleration Relative Power around 8Hz [] | Frequency | 0.15 0.15 0.08 0.14 | 4.66e-05 1.09e-04 4.89e-02 6.05e-04 | Weak Weak Weak Weak |
| 3D Acceleration Relative Power around 11Hz [] | Frequency | 0.17 0.33 0.11 0.31 | 8.02e-06 5.35e-19 3.83e-03 9.73e-16 | Weak Moderate Weak Moderate |
| Acceleration Tremor Principal Component Relative Power in the Band of Dyskinetic Motion [] | Frequency | -0.38 -0.56 -0.22 -0.42 | 2.59e-25 3.95e-58 3.54e-08 9.95e-28 | Moderate Moderate Weak Moderate |
| Acceleration Tremor Principal Component Relative Power in the Band of Parkinsonian Tremor [] | Frequency | -0.03 0.08 -0.09 -0.01 | 2.58e-02 1.82e-02 | Weak Weak |
| Acceleration Tremor Principal Component Relative Power in the Band of Physiological Tremor [] | Frequency | 0.31 0.39 0.17 0.34 | 1.44e-17 3.34e-27 1.89e-05 4.17e-18 | Moderate Moderate Weak Moderate |
| Acceleration Tremor Principal Component Relative Power around 2Hz [] | Frequency | -0.37 -0.54 -0.12 -0.35 | 6.38e-24 1.09e-53 1.83e-03 3.78e-20 | Moderate Moderate Weak Moderate |
| Acceleration Tremor Principal Component Relative Power around 5Hz [] | Frequency | -0.03 0.08 0.02 0.06 | 3.02e-02 | Weak |
| Acceleration Tremor Principal Component Relative Power around 8Hz [] | Frequency | 0.14 0.10 0.03 0.14 | 1.27e-04 8.63e-03  3.45e-04 | Weak Weak  Weak |
| Acceleration Tremor Principal Component Relative Power around 11Hz [] | Frequency | 0.35 0.46 0.18 0.35 | 9.08e-22 1.90e-38 8.70e-06 1.03e-19 | Moderate Moderate Weak Moderate |
| Dominant Acceleration Tremor Relative Power in the Band of Dyskinetic Motion [] | Frequency | -0.32 -0.50 -0.09 -0.32 | 4.87e-18 1.94e-45 2.56e-02 6.46e-17 | Moderate Moderate Weak Moderate |
| Dominant Acceleration Tremor Relative Power in the Band of Parkinsonian Tremor [] | Frequency | 0.03 0.12 -0.03 0.01 | 1.08e-03 | Weak |
| Dominant Acceleration Tremor Relative Power in the Band of Physiological Tremor [] | Frequency | 0.27 0.34 0.18 0.36 | 1.79e-13 7.59e-21 6.89e-06 7.50e-21 | Weak Moderate Weak Moderate |
| Dominant Acceleration Tremor Relative Power around 2Hz [] | Frequency | -0.40 -0.53 -0.17 -0.38 | 2.90e-28 2.75e-52 1.13e-05 1.00e-22 | Moderate Moderate Weak Moderate |
| Dominant Acceleration Tremor Relative Power around 5Hz [] | Frequency | 0.06 0.13 0.09 0.08 | 6.11e-04 2.76e-02 | Weak Weak |
| Dominant Acceleration Tremor Relative Power around 8Hz [] | Frequency | 0.09 0.07 0.01 0.12 | 2.13e-02   1.80e-03 | Weak   Weak |
| Dominant Acceleration Tremor Relative Power around 11Hz [] | Frequency | 0.32 0.42 0.18 0.38 | 1.62e-18 1.18e-30 5.45e-06 3.14e-23 | Moderate Moderate Weak Moderate |
| Angular Velocity Tremor Principal Component Relative Power in the Band of Dyskinetic Motion [] | Frequency | -0.52 -0.45 -0.36 -0.34 | 9.65e-49 3.53e-36 2.96e-21 8.51e-19 | Moderate Moderate Moderate Moderate |
| Angular Velocity Tremor Principal Component Relative Power in the Band of Parkinsonian Tremor [] | Frequency | 0.43 0.37 0.23 0.19 | 6.23e-33 2.05e-24 2.25e-09 1.16e-06 | Moderate Moderate Weak Weak |
| Angular Velocity Tremor Principal Component Relative Power in the Band of Physiological Tremor [] | Frequency | 0.06 0.11 -0.04 -0.04 | 2.68e-03 | Weak |
| Angular Velocity Tremor Principal Component Relative Power around 2Hz [] | Frequency | -0.48 -0.41 -0.28 -0.24 | 2.17e-42 4.74e-30 3.53e-13 2.16e-09 | Moderate Moderate Weak Weak |
| Angular Velocity Tremor Principal Component Relative Power around 5Hz [] | Frequency | 0.49 0.42 0.34 0.32 | 4.79e-44 4.71e-32 4.18e-19 2.44e-16 | Moderate Moderate Moderate Moderate |
| Angular Velocity Tremor Principal Component Relative Power around 8Hz [] | Frequency | 0.07 0.06 -0.04 -0.04 |  |  |
| Angular Velocity Tremor Principal Component Relative Power around 11Hz [] | Frequency | 0.09 0.17 -0.00 0.01 | 1.33e-02 5.94e-06 | Weak Weak |
| Dominant Angular Velocity Tremor Relative Power in the Band of Dyskinetic Motion [] | Frequency | -0.42 -0.35 -0.14 -0.15 | 1.76e-31 8.81e-22 6.25e-04 1.78e-04 | Moderate Moderate Weak Weak |
| Dominant Angular Velocity Tremor Relative Power in the Band of Parkinsonian Tremor [] | Frequency | 0.46 0.38 0.21 0.21 | 1.54e-37 8.31e-25 1.46e-07 8.84e-08 | Moderate Moderate Weak Weak |
| Dominant Angular Velocity Tremor Relative Power in the Band of Physiological Tremor [] | Frequency | 0.04 0.09 -0.09 -0.07 | 1.50e-02 1.99e-02 | Weak Weak k |
| Dominant Angular Velocity Tremor Relative Power around 2Hz [] | Frequency | -0.51 -0.39 -0.26 -0.26 | 1.47e-47 3.71e-27 2.69e-11 7.24e-11 | Moderate Moderate Weak Weak |
| Dominant Angular Velocity Tremor Relative Power around 5Hz [] | Frequency | 0.53 0.41 0.34 0.34 | 8.86e-53 7.82e-30 3.25e-19 3.61e-18 | Moderate Moderate Moderate Moderate |
| Dominant Angular Velocity Tremor Relative Power around 8Hz [] | Frequency | 0.04 0.07 -0.09 -0.04 | 2.73e-02 | Weak |
| Dominant Angular Velocity Tremor Relative Power around 11Hz [] | Frequency | 0.08 0.13 -0.06 -0.03 | 2.70e-02 6.31e-04 | Weak Weak |
| 3D Acceleration Spectral Peak Frequency [Hz] | Frequency | 0.04 0.05 0.01 0.09 | 2.74e-02 | Weak |
| Acceleration Tremor Principal Component Spectral Peak Frequency [Hz] | Frequency | 0.13 0.21 -0.01 0.10 | 3.90e-04 1.37e-08  8.35e-03 | Weak Weak  Weak |
| Dominant Acceleration Tremor Spectral Peak Frequency [Hz] | Frequency | 0.11 0.21 -0.02 0.05 | 4.71e-03 1.09e-08 | Weak Weak |
| Angular Velocity Tremor Principal Component Spectral Peak Frequency [Hz] | Frequency | 0.50 0.34 0.33 0.29 | 3.36e-46 7.00e-21 1.21e-17 4.65e-14 | Moderate Moderate Moderate Weak |
| Dominant Angular Velocity Tremor Spectral Peak Frequency [Hz] | Frequency | 0.51 0.38 0.32 0.32 | 8.96e-47 6.46e-26 1.48e-16 2.97e-16 | Moderate Moderate Moderate Moderate |
| 3D Acceleration Spectral Peak Amplitude [(mm/s^2)^2/Hz] | Frequency | -0.00 0.03 0.20 0.07 | 6.68e-07 | Weak |
| Acceleration Tremor Principal Component Spectral Peak Amplitude [(mm/s^2)^2/Hz] | Frequency | 0.38 0.42 0.29 0.37 | 1.36e-25 1.17e-31 2.82e-14 6.26e-22 | Moderate Moderate Weak Moderate |
| Dominant Acceleration Tremor Spectral Peak Amplitude [(mm/s^2)^2/Hz] | Frequency | 0.39 0.42 0.31 0.38 | 5.04e-27 6.75e-32 4.84e-16 9.53e-24 | Moderate Moderate Moderate Moderate |
| Angular Velocity Tremor Principal Component Spectral Peak Amplitude [(deg/s)^2/Hz] | Frequency | 0.23 0.23 0.15 0.21 | 1.09e-09 1.74e-09 9.61e-05 9.52e-08 | Weak Weak Weak Weak |
| Dominant Angular Velocity Tremor Spectral Peak Amplitude [(deg/s)^2/Hz] | Frequency | 0.21 0.21 0.13 0.18 | 1.10e-08 2.35e-08 1.37e-03 5.63e-06 | Weak Weak Weak Weak |
| Dominant Acceleration Tremor Amplitude Modulated Outlier Level [(mm/s^2)^2/Hz] | Frequency | 0.40 0.36 0.33 0.39 | 3.22e-28 4.88e-23 8.28e-18 2.89e-24 | Moderate Moderate Moderate Moderate |
| Dominant Acceleration Tremor Outlier Level [] | Frequency | 0.31 0.20 0.14 0.13 | 1.00e-16 8.49e-08 6.16e-04 9.15e-04 | Moderate Weak Weak Weak |
| Dominant Acceleration Tremor Peak Power Concentration 68% [Hz] | Frequency | 0.15 0.19 0.06 0.18 | 6.03e-05 8.08e-07  7.74e-06 | Weak Weak  Weak |
| Dominant Acceleration Tremor Peak Power Concentration 50% [Hz] | Frequency | 0.05 0.00 -0.03 0.05 |  |  |
| Dominant Acceleration Tremor Sorted 68% Bandwidth Frequency [Hz] | Frequency | 0.05 0.16 0.06 0.21 | 1.88e-05  8.31e-08 | Weak  Weak |
| Dominant Acceleration Tremor Sorted Median Frequency [Hz] | Frequency | -0.02 0.08 0.01 0.16 | 3.39e-02  4.24e-05 | Weak  Weak |
| Dominant Acceleration Tremor Sorted Centre of Mass Frequency [Hz] | Frequency | 0.02 0.13 0.05 0.21 | 3.86e-04  1.18e-07 | Weak  Weak |
| 3D Acceleration Amplitude Modulated Outlier Level [(mm/s^2)^2/Hz] | Frequency | 0.13 0.01 0.10 0.06 | 5.28e-04  1.00e-02 | Weak  Weak |
| 3D Acceleration Outlier Level [] | Frequency | 0.01 -0.20 -0.08 -0.15 | 7.80e-08 4.70e-02 2.03e-04 | Weak Weak Weak |
| 3D Acceleration Peak Power Concentration 68% [Hz] | Frequency | 0.13 0.26 0.12 0.23 | 3.72e-04 1.12e-12 3.00e-03 5.16e-09 | Weak Weak Weak Weak |
| 3D Acceleration Peak Power Concentration 50% [Hz] | Frequency | 0.21 0.32 0.08 0.25 | 1.23e-08 3.76e-18 3.95e-02 3.77e-10 | Weak Moderate Weak Weak |
| 3D Acceleration Sorted 68% Bandwidth Frequency [Hz] | Frequency | 0.14 0.30 0.15 0.31 | 2.36e-04 2.99e-16 1.40e-04 1.35e-15 | Weak Moderate Weak Moderate |
| 3D Acceleration Sorted Median Frequency [Hz] | Frequency | 0.14 0.34 0.14 0.32 | 1.97e-04 6.71e-20 6.04e-04 2.30e-16 | Weak Moderate Weak Moderate |
| 3D Acceleration Sorted Centre of Mass Frequency [Hz] | Frequency | 0.14 0.33 0.15 0.34 | 1.38e-04 8.18e-20 1.48e-04 6.14e-19 | Weak Moderate Weak Moderate |
| Acceleration Tremor Principal Component Amplitude Modulated Outlier Level [(mm/s^2)^2/Hz] | Frequency | 0.33 0.31 0.25 0.32 | 8.05e-20 1.34e-16 2.83e-10 6.09e-17 | Moderate Moderate Weak Moderate |
| Acceleration Tremor Principal Component Outlier Level [] | Frequency | 0.34 0.25 0.07 0.08 | 2.53e-20 1.65e-11  3.98e-02 | Moderate Weak  Weak |
| Acceleration Tremor Principal Component Peak Power Concentration 68% [Hz] | Frequency | 0.22 0.20 0.07 0.23 | 1.93e-09 8.09e-08  6.11e-09 | Weak Weak  Weak |
| Acceleration Tremor Principal Component Peak Power Concentration 50% [Hz] | Frequency | 0.13 0.04 -0.01 0.11 | 3.48e-04   5.42e-03 | Weak   Weak |
| Acceleration Tremor Principal Component Sorted 68% Bandwidth Frequency [Hz] | Frequency | 0.13 0.19 0.10 0.23 | 4.97e-04 4.60e-07 1.29e-02 6.68e-09 | Weak Weak Weak Weak |
| Acceleration Tremor Principal Component Sorted Median Frequency [Hz] | Frequency | 0.05 0.12 0.05 0.18 | 9.55e-04  5.51e-06 | Weak  Weak |
| Acceleration Tremor Principal Component Sorted Centre of Mass Frequency [Hz] | Frequency | 0.09 0.17 0.08 0.22 | 1.20e-02 4.08e-06  1.75e-08 | Weak Weak  Weak |
| Dominant Angular Velocity Tremor Amplitude Modulated Outlier Level [(deg/s)^2/Hz] | Frequency | 0.36 0.12 0.34 0.29 | 1.46e-22 1.31e-03 3.18e-19 8.13e-14 | Moderate Weak Moderate Weak |
| Dominant Angular Velocity Tremor Outlier Level [] | Frequency | 0.26 -0.03 0.18 -0.03 | 2.85e-12  3.81e-06 | Weak  Weak |
| Dominant Angular Velocity Tremor Peak Power Concentration 68% [Hz] | Frequency | 0.04 0.22 -0.12 -0.02 | 5.64e-09 1.91e-03 | Weak Weak |
| Dominant Angular Velocity Tremor Peak Power Concentration 50% [Hz] | Frequency | -0.05 0.21 -0.15 -0.00 | 3.06e-08 1.05e-04 | Weak Weak |
| Dominant Angular Velocity Tremor Sorted 68% Bandwidth Frequency [Hz] | Frequency | -0.08 0.16 -0.17 -0.02 | 3.28e-02 1.46e-05 2.22e-05 | Weak Weak Weak |
| Dominant Angular Velocity Tremor Sorted Median Frequency [Hz] | Frequency | -0.13 0.16 -0.18 -0.01 | 5.02e-04 1.26e-05 8.63e-06 | Weak Weak Weak |
| Dominant Angular Velocity Tremor Sorted Centre of Mass Frequency [Hz] | Frequency | -0.08 0.20 -0.16 0.02 | 2.99e-02 5.22e-08 4.93e-05 | Weak Weak Weak |
| Angular Velocity Tremor Principal Component Amplitude Modulated Outlier Level [(deg/s)^2/Hz] | Frequency | 0.16 -0.03 0.08 -0.01 | 2.24e-05  4.61e-02 | Weak  Weak |
| Angular Velocity Tremor Principal Component Outlier Level [] | Frequency | 0.11 -0.07 -0.02 -0.14 | 5.37e-03   2.65e-04 | Weak   Weak |
| Angular Velocity Tremor Principal Component Peak Power Concentration 68% [Hz] | Frequency | 0.10 0.09 -0.01 -0.01 | 6.87e-03 1.43e-02 | Weak Weak |
| Angular Velocity Tremor Principal Component Peak Power Concentration 50 [Hz] | Frequency | 0.01 0.10 -0.02 -0.03 | 1.19e-02 | Weak |
| Angular Velocity Tremor Principal Component Sorted 68% Bandwidth Frequency [Hz] | Frequency | 0.01 0.10 -0.03 -0.01 | 6.90e-03 | Weak |
| Angular Velocity Tremor Principal Component Sorted Median Frequency [Hz] | Frequency | -0.02 0.12 -0.01 0.02 | 1.13e-03 | Weak |
| Angular Velocity Tremor Principal Component Sorted Centre of Mass Frequency [Hz] | Frequency | 0.00 0.12 -0.02 -0.00 | 1.93e-03 | Weak |
| 3D Acceleration 90% Bandwidth Frequency [Hz] | Frequency | 0.49 0.54 0.38 0.54 | 9.38e-43 2.33e-53 5.89e-23 8.52e-49 | Moderate Moderate Moderate Moderate |
| Acceleration Tremor First Principal Component 90% Bandwidth Frequency [Hz] | Frequency | 0.38 0.47 0.22 0.40 | 5.81e-26 1.62e-39 2.57e-08 3.37e-26 | Moderate Moderate Weak Moderate |
| Acceleration Tremor First and Second Principal Components 90% Bandwidth Frequency [Hz] | Frequency | 0.34 0.47 0.21 0.36 | 5.15e-20 7.32e-39 1.29e-07 3.04e-20 | Moderate Moderate Weak Moderate |
| Acceleration Tremor Principal Component 90% Bandwidth Frequency [Hz] | Frequency | 0.34 0.45 0.17 0.35 | 5.86e-21 6.12e-37 1.15e-05 3.71e-19 | Moderate Moderate Weak Moderate |
| Dominant Acceleration Tremor 90% Bandwidth Frequency [Hz] | Frequency | 0.31 0.40 0.21 0.40 | 2.18e-17 1.74e-28 7.69e-08 4.11e-25 | Moderate Moderate Weak Moderate |
| Median Acceleration 90% Bandwidth Frequency [Hz] | Frequency | 0.45 0.53 0.36 0.47 | 1.72e-35 7.40e-53 2.16e-20 2.16e-36 | Moderate Moderate Moderate Moderate |
| Median Acceleration Tremor 90% Bandwidth Frequency [Hz] | Frequency | 0.35 0.52 0.25 0.39 | 9.78e-22 6.38e-49 1.94e-10 9.63e-25 | Moderate Moderate Weak Moderate |
| 3D Acceleration Median Frequency [Hz] | Frequency | 0.40 0.41 0.30 0.38 | 1.49e-28 1.63e-30 2.66e-14 3.13e-23 | Moderate Moderate Weak Moderate |
| Acceleration Tremor First Principal Component Median Frequency [Hz] | Frequency | 0.28 0.32 0.11 0.29 | 9.30e-14 2.84e-18 5.28e-03 5.25e-14 | Weak Moderate Weak Weak |
| Acceleration Tremor First and Second Principal Components Median Frequency [Hz] | Frequency | 0.25 0.32 0.06 0.27 | 1.65e-11 5.98e-18  8.69e-12 | Weak Moderate  Weak |
| Acceleration Tremor Principal Component Median Frequency [Hz] | Frequency | 0.26 0.33 0.06 0.27 | 5.27e-12 7.39e-19  4.16e-12 | Weak Moderate k Weak |
| Dominant Acceleration Tremor Median Frequency [Hz] | Frequency | 0.20 0.28 0.08 0.27 | 6.73e-08 7.58e-14  3.06e-12 | Weak Weak  Weak |
| Median Acceleration Median Frequency [Hz] | Frequency | 0.31 0.35 0.22 0.28 | 4.11e-17 1.32e-21 3.85e-08 4.23e-13 | Moderate Moderate Weak Weak |
| Median Acceleration Tremor Median Frequency [Hz] | Frequency | 0.32 0.41 0.11 0.31 | 6.85e-18 3.92e-30 5.07e-03 1.48e-15 | Moderate Moderate Weak Moderate |
| 3D Angular Velocity 90% Bandwidth Frequency [Hz] | Frequency | 0.60 0.54 0.29 0.35 | 2.71e-69 2.66e-54 9.15e-14 1.68e-19 | Moderate Moderate Weak Moderate |
| Angular Velocity Tremor First Principal Component 90% Bandwidth Frequency [Hz] | Frequency | 0.03 0.09 -0.02 -0.01 | 1.22e-02 | Weak |
| Angular Velocity Tremor First and Second Principal Component 90% Bandwidth Frequency [Hz] | Frequency | 0.10 0.13 -0.03 -0.01 | 6.58e-03 7.16e-04 | Weak Weak |
| Angular Velocity Tremor Principal Component 90% Bandwidth Frequency [Hz] | Frequency | 0.08 0.11 -0.03 -0.03 | 2.88e-02 2.56e-03 | Weak Weak |
| Dominant Angular Velocity Tremor 90% Bandwidth Frequency [Hz] | Frequency | 0.07 0.10 -0.09 -0.05 | 8.50e-03 2.54e-02 | Weak Weak |
| Median Angular Velocity Tremor 90% Bandwidth Frequency [Hz] | Frequency | 0.51 0.55 0.33 0.42 | 2.30e-48 3.86e-57 1.93e-17 1.91e-28 | Moderate Moderate Moderate Moderate |
| Median Angular Velocity Tremor 90% Bandwidth Frequency [Hz] | Frequency | 0.17 0.29 0.04 0.07 | 4.49e-06 9.84e-15 | Weak Weak |
| 3D Angular Velocity Median Frequency [Hz] | Frequency | 0.59 0.50 0.42 0.51 | 1.71e-67 7.28e-46 2.71e-28 5.15e-44 | Moderate Moderate Moderate Moderate |
| Angular Velocity Tremor First Principal Component Median Frequency [Hz] | Frequency | 0.40 0.33 0.23 0.18 | 7.69e-29 1.04e-19 7.38e-09 9.07e-06 | Moderate Moderate Weak Weak |
| Angular Velocity Tremor First and Second Principal Component Median Frequency [Hz] | Frequency | 0.43 0.40 0.21 0.20 | 9.01e-33 4.90e-28 4.91e-08 6.54e-07 | Moderate Moderate Weak Weak |
| Angular Velocity Tremor Principal Component Median Frequency [Hz] | Frequency | 0.41 0.37 0.22 0.18 | 4.83e-30 1.15e-24 2.81e-08 5.48e-06 | Moderate Moderate Weak Weak |
| Dominant Angular Velocity Tremor Median Frequency [Hz] | Frequency | 0.44 0.36 0.20 0.20 | 1.50e-34 1.87e-23 2.82e-07 2.90e-07 | Moderate Moderate Weak Weak |
| Median Angular Velocity Median Frequency [Hz] | Frequency | 0.72 0.73 0.62 0.69 | 8.61e-114 6.07e-117 1.33e-69 3.54e-90 | Strong Strong Moderate Moderate |
| Median Angular Velocity Tremor Median Frequency [Hz] | Frequency | 0.57 0.62 0.32 0.32 | 1.92e-60 2.75e-74 4.07e-17 4.80e-16 | Moderate Moderate Moderate Moderate |
| Tilt 90% Bandwidth Frequency [Hz] | Frequency | 0.28 0.49 0.02 0.36 | 8.31e-14 2.92e-43  8.89e-21 | Weak Moderate  Moderate |
| Tilt Median Frequency [Hz] | Frequency | 0.09 0.39 0.11 0.32 | 1.44e-02 5.48e-27 3.95e-03 3.97e-16 | Weak Moderate Weak Moderate |
| Median Angular Velocity Tremor Relative Power around the Peak Frequency Mode [] | Frequency | -0.01 -0.20 0.06 0.00 | 4.84e-08 | Weak |
| Mean Angular Velocity Tremor Relative Power around the Peak Frequency Mode [] | Frequency | 0.01 -0.20 0.09 -0.01 | 1.87e-07 1.66e-02 | Weak Weak |
| Angular Velocity Tremor Principal Component Relative Power around the Peak [] | Frequency | 0.07 -0.05 0.05 0.07 | 4.80e-02 | Weak |
