# Supplementary figures and images for: Quantitative analysis of handwriting kinematics in primary and lower secondary school children through a sensorized ink pen: A cross-sectional population-based study
Source: PLOS Digit Health. 2026 Jul 23;5(7):e0001503. doi: 10.1371/journal.pdig.0001503 (PMC13395322; doi:10.1371/journal.pdig.0001503)

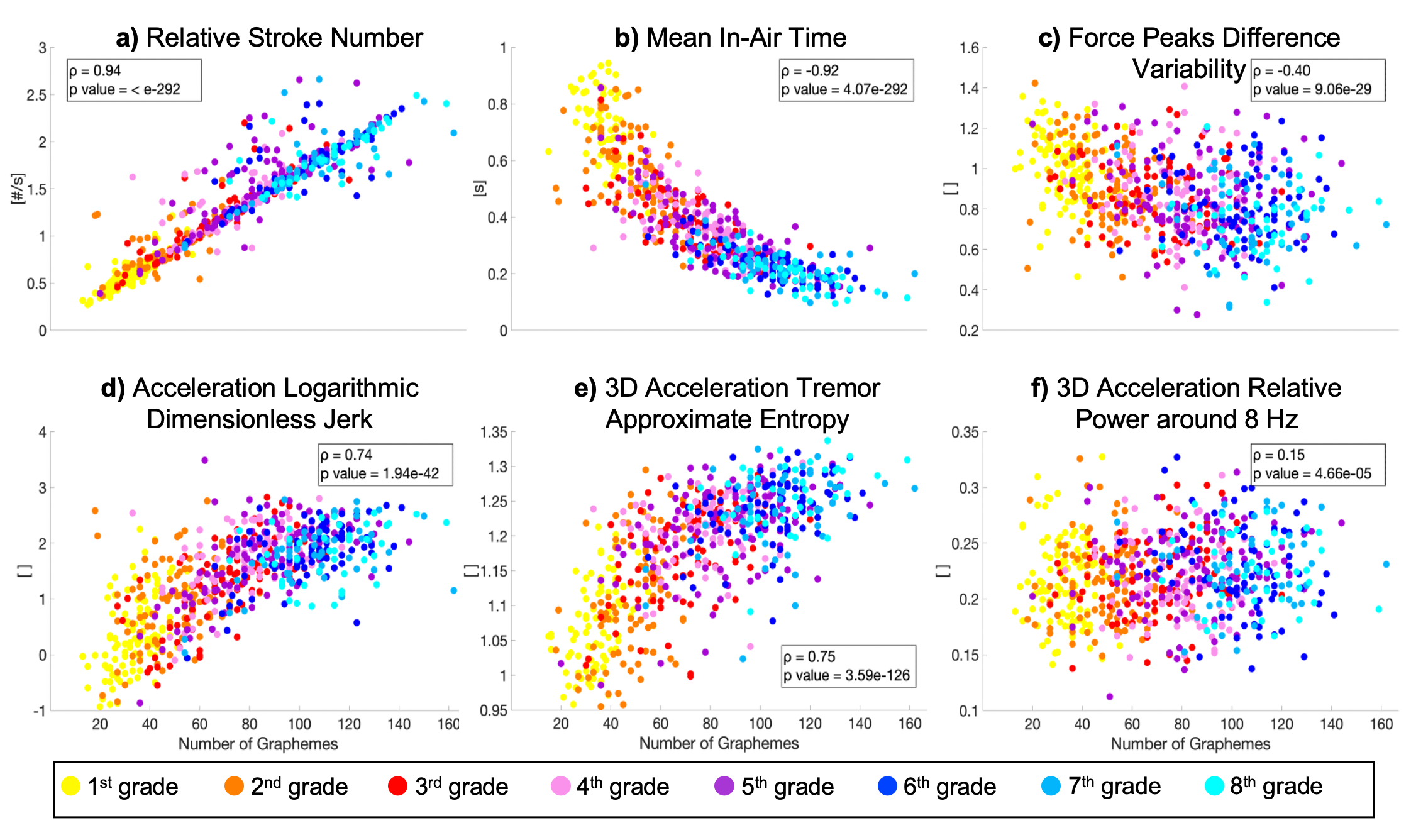

Supplement: S1 Fig — Scatterplots for UNO_B, with correlation coefficient (ρ) and p value. Yellow: 1st grade T1. Orange: 2nd grade T1. Red: 3rd grade. Pink: 4th grade. Purple: 5th grade. Blue: 6th grade. Light blue: 7th grade. Cyan: 8th grade. (TIF) [file pdig.0001503.s003.tif]

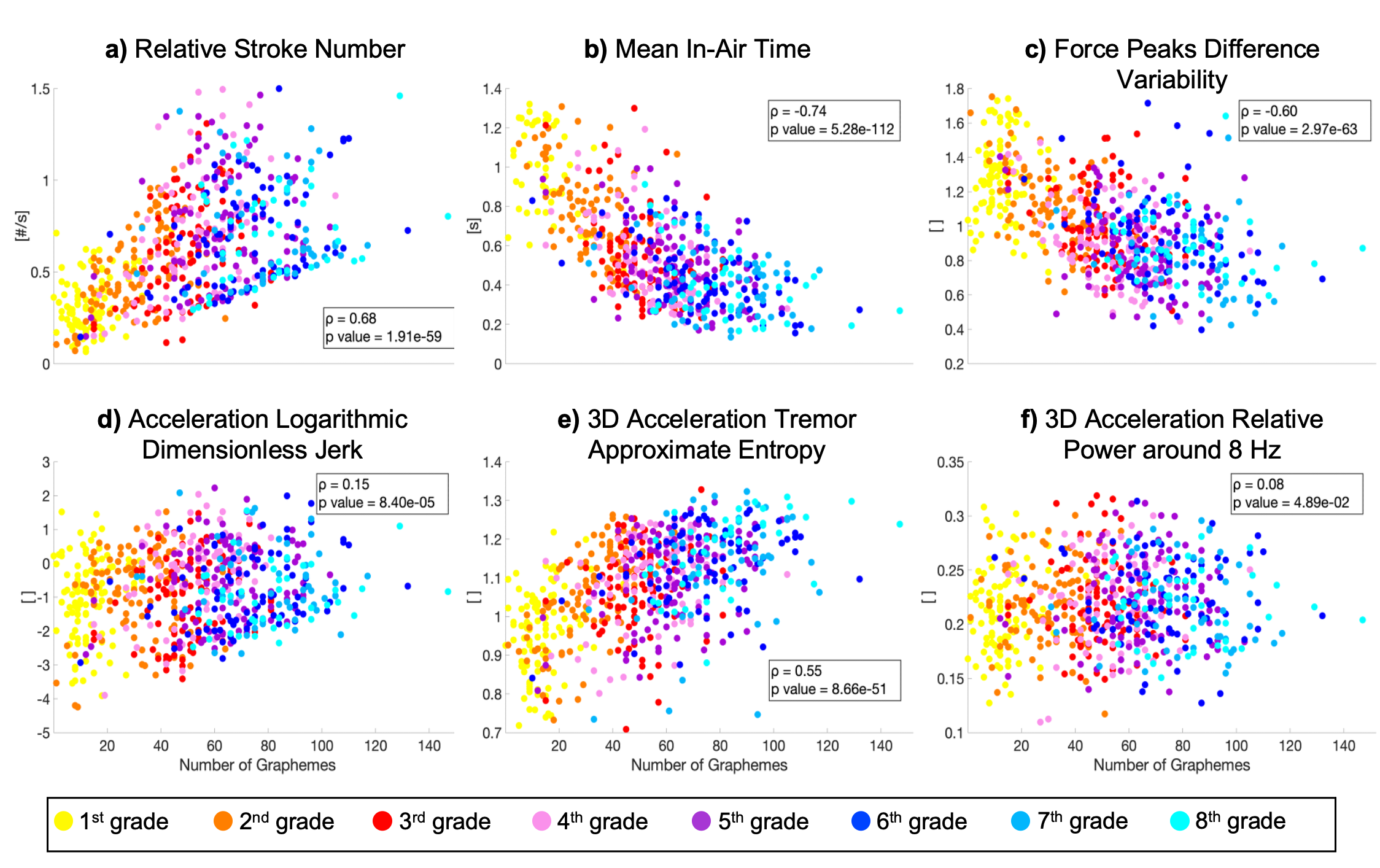

Supplement: S2 Fig — Scatterplots for UNO_c, with correlation coefficient (ρ) and p value. Yellow: 1st grade T1. Orange: 2nd grade T1. Red: 3rd grade. Pink: 4th grade. Purple: 5th grade. Blue: 6th grade. Light blue: 7th grade. Cyan: 8th grade. (TIF) [file pdig.0001503.s004.tif]

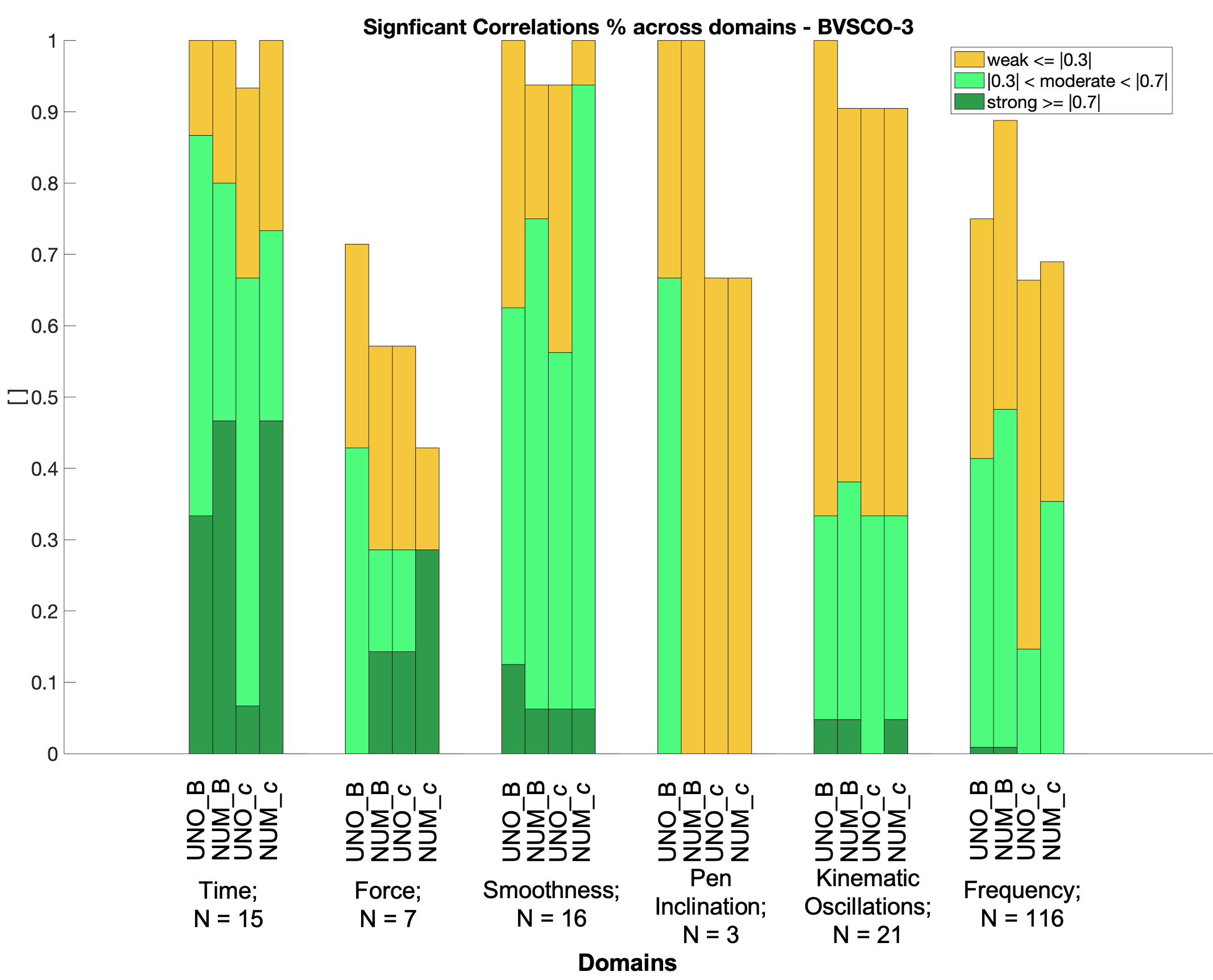

Supplement: S3 Fig — Dark Green: Strong Correlation; Light Green: Moderate Correlation; Orange: Weak Correlation. The vertical axis is normalized according to the total number of indicators in the domain (N). (TIFF) [file pdig.0001503.s005.tiff]
